# Microbial and Chemical Characterization of Underwater Fresh Water Springs in the Dead Sea

**DOI:** 10.1371/journal.pone.0038319

**Published:** 2012-06-05

**Authors:** Danny Ionescu, Christian Siebert, Lubos Polerecky, Yaniv Y. Munwes, Christian Lott, Stefan Häusler, Mina Bižić-Ionescu, Christian Quast, Jörg Peplies, Frank Oliver Glöckner, Alban Ramette, Tino Rödiger, Thorsten Dittmar, Aharon Oren, Stefan Geyer, Hans-Joachim Stärk, Martin Sauter, Tobias Licha, Jonathan B. Laronne, Dirk de Beer

**Affiliations:** 1 Max Planck Institute for Marine Microbiology, Bremen, Germany; 2 Department of Catchment Hydrology, Helmholtz-Centre for Environmental Research — UFZ, Halle/Saale, Germany; 3 Department of Geography & Environmental Development, Ben Gurion University of the Negev, Beer Sheva, Israel; 4 HYDRA Institute for Marine Sciences, Elba Field Station, Campo nell’Elba (LI), Italy; 5 Department of Stratified Lakes, Leibniz Institute of Freshwater Ecology and Inland Fisheries Berlin, Stechlin, Germany; 6 Ribocon GmbH, Bremen, Germany; 7 Jacobs University Bremen GmbH, Bremen, Germany; 8 Institute for Chemistry and Biology of the Marine Environment, Carl von Ossietzky University, Oldenburg, Germany; 9 Department of Plant and Environmental Sciences, Institute of Life Sciences, The Hebrew University of Jerusalem, Jerusalem, Israel; 10 Department of Analytical Chemistry, Helmholtz-Centre for Environmental Research – UFZ, Leipzig, Germany; 11 Geoscientific Centre, University of Göttingen, Göttingen, Germany; Uppsala University, Sweden

## Abstract

Due to its extreme salinity and high Mg concentration the Dead Sea is characterized by a very low density of cells most of which are Archaea. We discovered several underwater fresh to brackish water springs in the Dead Sea harboring dense microbial communities. We provide the first characterization of these communities, discuss their possible origin, hydrochemical environment, energetic resources and the putative biogeochemical pathways they are mediating. Pyrosequencing of the 16S rRNA gene and community fingerprinting methods showed that the spring community originates from the Dead Sea sediments and not from the aquifer. Furthermore, it suggested that there is a dense Archaeal community in the shoreline pore water of the lake. Sequences of bacterial sulfate reducers, nitrifiers iron oxidizers and iron reducers were identified as well. Analysis of white and green biofilms suggested that sulfide oxidation through chemolitotrophy and phototrophy is highly significant. Hyperspectral analysis showed a tight association between abundant green sulfur bacteria and cyanobacteria in the green biofilms. Together, our findings show that the Dead Sea floor harbors diverse microbial communities, part of which is not known from other hypersaline environments. Analysis of the water’s chemistry shows evidence of microbial activity along the path and suggests that the springs supply nitrogen, phosphorus and organic matter to the microbial communities in the Dead Sea. The underwater springs are a newly recognized water source for the Dead Sea. Their input of microorganisms and nutrients needs to be considered in the assessment of possible impact of dilution events of the lake surface waters, such as those that will occur in the future due to the intended establishment of the Red Sea−Dead Sea water conduit.

## Introduction

The Dead Sea is a terminal lake located on the border between Jordan, the Palestinian Authority and Israel, and is part of a larger geological system known as the Jordan Dead Sea Rift. The lake consists of a deeper northern basin (deepest point at ∼725 m below sea level) and a southern basin, which has dried out but is kept shallow by continuous transfer of water from the northern basin as it is used for commercial mineral production. Until 1979 the Dead Sea was a meromictic lake with hypersaline, anoxic and sulfidic deep waters and a seasonally varying mixolimnion [1]. Since the beginning of the 20th century the water budget of the Dead Sea has been negative, leading to a continuous decrease in the water level [1], [2]. The extensive evaporation in the absence of major water input led to an increase in the density of the upper water layer, which caused the lake to overturn in 1979 [3] Since then, except after two rainy seasons in 1980 and 1992, the Dead Sea remained holomictic and has been characterized by a NaCl supersaturation and halite deposition on the lake bottom, with total dissolved salt concentrations reaching 347 g L^−1^. Due to the continuous evaporation of the Dead Sea, Na^+^ precipitates out as halite while Mg^2+^,whose salts are more soluble, is further concentrated and has become the dominant cation [2].

The increased salinity and the elevated concentration of divalent ions make the Dead Sea an extreme environment that is not tolerated by most organisms. This is reflected in a generally low diversity and very low abundance of microorganisms. The microbiology of the lake has been subject for research since the 1930s when Benjamin Elazari-Volcani (Wilkansky at the time) isolated the first microorganisms from the sediment of the Dead Sea [4]. Besides Bacteria and Archaea [5] these isolates included algae [6], protozoa [7], and ciliates [8]. Since then, several Bacterial and Archaeal isolates have been obtained in culture, both from the sediment and from the water body [2]. The general cell abundance in the Dead Sea water is very low (<5×10^4^ cells mL^−1^; this study), except for two blooms in 1980 and 1992, when after severe winters the upper meter of the water column was diluted by 15–30%, floods provided an input of phosphate, and the cell concentrations reached 20–35×10^6^ cells mL^−1^ [9].

Recently, we discovered a complex system of underwater springs in the Dead Sea. A more detailed exploration revealed that these springs harbor microbial communities with much higher diversity and cell density than reported to date for the Dead Sea, including dense biofilms covering sediments and rocks around the springs. In this study we provide the first description of these habitats and the associated microbial communities. Based on comparative analyses of the community structure and geochemical reconstruction of the spring water sources, we propose hypotheses about the main energy resources and metabolic pathways that drive these microbial ecosystems, as well as discuss their possible origins and environmental adaptations.

## Methods

### Site Description

**Figure 1:**
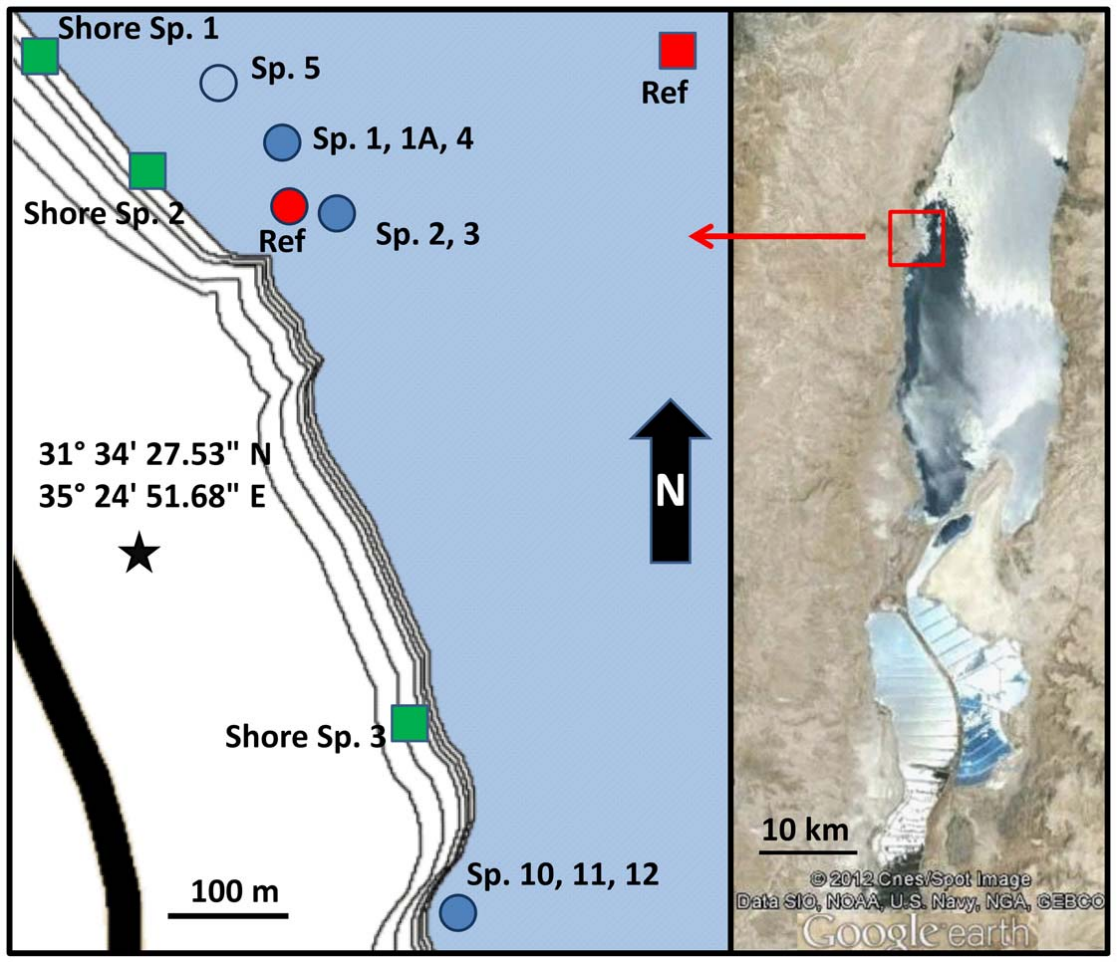
Locations of the sampling sites on the west coast of the Dead Sea, showing the northern and southern spring systems. Underwater springs with the corresponding reference site are marked with blue and red circles, whereas shore springs together with their reference site are marked with green and red squares, respectively. The open-water reference site for the shore springs was used only for comparison of dissolved organic matter (DOM) and total dissolved nitrogen (TDN). The open blue circle is located in the center of an underwater spring upwelling and was sampled for DOM and TDN analysis. The contour lines on the left panel represent the yearly drop in the lake level and are a close approximation of the areal topography. The satellite image was created using Google Earth.

**Figure 2:**
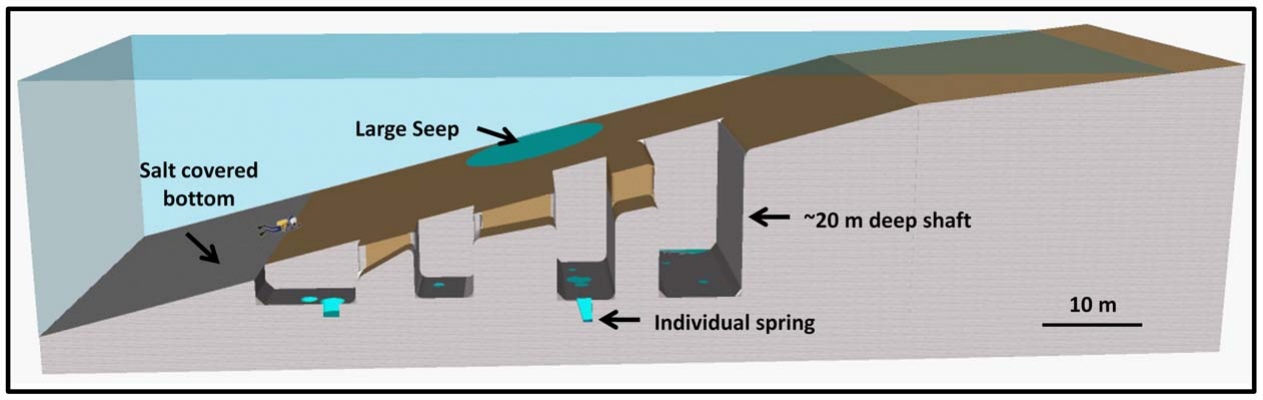
Sketch of the northern spring system. The water seep shown on the slope of the sketch is found only in deeper parts of the southern system where water seeps through the sediment surface over a large area without defined boundaries. The shafts have steep, laminated walls (see Fig. S1A) and contain one or more springs (blue). Localized water sources are either directly visible on the shaft bottom (Fig. S1B) or are hidden within deeper cavities (Fig. S1C). In the southern spring system (not shown in the sketch) springs do not form shafts and are covered by cobbles (Fig. S1D).

The underwater springs are located in the Darga area on the western coast of the Dead Sea, and are divided into two systems (Fig. 1). The northern system (springs 1–5) consists of one or more springs at the bottom of deep steep-walled shafts (Fig. 2A). Often several such shafts are connected and form a large system that extends from shallow (∼10 m) to deeper (∼30 m) waters. The diameter and depth of each shaft can reach up to 15 and 20 m, respectively. The walls of the shafts are finely laminated (Fig. S1A). Groundwater emerges from either small seeps (∼20 cm in diameter, Fig. S1B) or deeper shafts that are hidden within deeper cavities (Fig. S1C). The springs in the southern system (springs 10–12) do not form shafts. Instead, they are located at the bottom of steep walls descending directly from the water surface, and are covered by cobble (Fig. S1D). Uniquely in the southern system and in addition to the described springs, large water seeps without clear boundaries were also found, mostly at depths of more than 15 m. Detailed hydrological and geological maps of the area can be found in Laronne Ben-Itzhak and Gvritzman [10].

### Sampling

Sampling of underwater springs for the analysis of microbial diversity, cell counts, pigments and water chemistry took place in June 2010 (Table 1). Samples for sulfide, ammonium, and phosphate quantification as well as for the analysis of dissolved organic carbon (DOC) and total dissolved nitrogen (TDN) were additionally collected from the same springs in October 2011. Prior to sampling, springs were marked by SCUBA divers based on light refraction in the mixing zone of the groundwater and the ambient Dead Sea water. Sediment and microbial mat samples were collected by SCUBA divers in cores and sterile 50 mL tubes,. Water samples from all springs except spring 5 were collected using a 40 m long hose connected to a peristaltic pump. A type K thermocouple cable was attached on the hose and connected to a thermometer (TM-747D, Tenmars, Taiwan) on the boat. Based on the difference in temperature, the submerged end of the pumping hose was placed into the spring by a SCUBA diver. Samples were collected once the density of the pumped water was stable and significantly lower than that of the Dead Sea, allowing several minutes (2–3 hose volumes) for washing of the hose with the spring water to minimize chemical and bacterial cross contamination. The pumping speed was kept low to prevent uptake of sediments or of the ambient Dead Sea water. Approximately 10 L of water were pumped from each spring. A water sample from the underwater spring 5 was collected from the surface of the Dead Sea where the emerging spring water formed an upwelling due to its low density compared to the Dead Sea water. This sample is therefore to an unknown extent mixed with ambient Dead Sea water. To allow a comparison between the native Dead Sea microbial communities and those associated with the underwater springs, reference sediment samples were additionally collected from an area without groundwater seepage (Fig. 1 Red Circle).

**Table 1:** Type of samples collected from the different underwater springs and auxiliary sites, and of analyses preformed.

Site	Type of samples collected			Type of analysis			
	Water	Sediment	Biofilm	Pigments	FISH	Community*	Chemistry
Spring 1	**+**	**+**	**−**	**−**	**+**	**+/+**	**+**
Spring 1A	**+**	**+**	**−**	**−**	**+**	**+/+**	**+**
Spring 2	**+**	**+**	**+**	**−**	**+**	**+/+**	**+**
Spring 3	**+**	**+**	**+**	**−**	**+**	**+/+**	**+**
Spring 4	**−**	**+**	**−**	**−**	**−**	**−/+**	**−**
Spring 5	**+**	**−**	**−**	**−**	**−**	**−**	**+**
Spring 10	**+**	**+**	**+**	**−**	**+**	**+/+**	**+**
Spring 11	**+**	**+**	**+**	**−**	**+**	**+/+**	**+**
Spring 12	**−**	**−**	**+**	**+**	**−**	**+/+**	**−**
Shore Spring 1, 2, 3	**+**	**−**	**−**	**−**	**−**	**−**	**+**
Dead Sea Reference	**−**	**+**	**−**	**−**	**−**	**+**	**−**
Dead Sea Water	**+**	**−**	**−**	**−**	**−**	**−**	**+**
Auja 2, 4	**+**	**−**	**−**	**−**	**−**	**−**	**+**
Jericho 2, 5	**+**	**−**	**−**	**−**	**−**	**−**	**+**
Mitzpe Jericho 2	**+**	**−**	**−**	**−**	**−**	**−**	**+**
EinQilt 1, 2	**+**	**−**	**−**	**−**	**−**	**−**	**+**
Qedem brine	**+**	**−**	**−**	**−**	**−**	**−**	**+**
Porewater	**+**	**−**	**−**	**−**	**−**	**−**	**+**
Sample Code	**W_SP#**	**S_SP#**	**(W/G)B_SP#**				

The northern system (springs 1**–**5), southern system (springs 10–12) and auxiliary sites are shaded in light and dark grey respectively. Community analysis refers to 454 pyrosequencing and Automated Ribosomal Intergenic Spacer Analysis (ARISA), pigment analysis was done by hyper-spectral imaging. Auja and Jericho 5 wells represent waters from the Lower Judea Group Aquifer (Mekorot Co., personal comm.), whereas Mitzpe Jericho 2 and Qilt springs represent waters originating from the marly sequences of the Upper Judea Group Aquifer (Mekorot Co., personal comm.). Qedem brine represents ascending water from the deep thermal aquifer south of the sampling area [10]. Pore water was sampled at a depth of 0.7 m below surface next to shore spring 3. The sample code field refers to the naming of the respective sample in subsequent analyses. W, S, B stand for water, sediment and biofilm respectively; w/g refers to white/green biofilms; SP# refers to the identification number of the spring.

To reveal the possible origin of the underwater springs water, samples for water chemistry analysis were collected from these auxiliary sites (Table 1): freshwater wells from the Upper and Lower Judea Group Aquifer (JGA); brackish springs emerging at the shore close to the sampling site at distances 1−90 m from the Dead Sea shore line (shore sp. 1–3 in Fig. 1); and shore springs in the Qedem area south of the sampling site, discharging hot brines from Lower Cretaceous or even Jurassic strata [11]. Pore water from the Dead Sea Group (DSG) sediment next to the shore spring 3 was sampled at depth of 0.7 m, which was 0.3 m above the 2010 Dead Sea water level. The pore water was squeezed from a sediment core on site using a mechanical, stainless steel, screw press. Thus the pore water represents interstitial waters transported by gravity from the exposed DSG sediments towards the Dead Sea. The term Dead Sea Group sediments will be used here on to describe all sediments which at some point throughout the history of the Dead Sea, were covered by its water.

Dead Sea water for dissolved organic matter (DOC) and total dissolved nitrogen (TDN) analysis was sampled away from the shore (Fig. 1 Square ref). To prevent influence of coastal and underwater spring waters, the samples were collected from a depth of 5 m.

All samples were kept at 4°C until further processing. Water samples for DNA extraction were filtered within 12 h, and the filters were kept at **−**20°C until further analysis, whereas sediments and microbial mats were transferred to **−**20°C within 24 h. The samples were transferred to Germany on dry ice for further processing.

Samples for water chemistry analysis were transported within 10 h of sampling to storage and 4°C; however, brine samples were stored at ambient conditions to prevent salt crystallization. Storage conditions were maintained during shipping to Germany for further analysis. Samples for cation analysis were pre acidified on site.

### Water Chemistry Analysis

Water (4 L) and pore water (0.6 L) samples were filtered on site through 0.22 µm cellulose-acetate filters and filled into separate bottles for cation and anion analyses. Cation analysis was conducted as previously described [12]. Shortly, the samples were additionally acidified by adding 0.3 mL of 6 M HCl. Determination of Mg, Ca, Sr, Rb, Cs, Mn was done by ICP-MS (Elan-DRC, Perkin Elmer, Germany)., whereas Na, K, Ba, B, Li, Si were analyzed with ICP-AES (Spectro Ciros CCD, Spectro Analytical Instruments,Germany)Both analyses were calibrated with matrix-adjusted standard solutions. Cl, Br and SO_4_ were analyzed by ion chromatography. HCO_3_^-^ was Gran-titrated adjusting the waters to pH 4.3 with H_2_SO_4_. For interpretation, ion concentrations were normalized to those in seawater, since seawater is a major source of brines and evaporates. H_2_S was analyzed colorimetrically using the methylene blue method [13]. pH temperature and Eh were measured on site, with a SenTix41 gel and SenTix platinum electrode, respectively connected to a WTW 350i field meter.

### Analysis of Dissolved Organic Carbon (DOC) and Total Dissolved Nitrogen (TDN)

DOC and TDN were analyzed in samples (250 mL) collected in acid-cleaned polycarbonate bottles, which were stored at **−**20°C until analysis at the Max Planck Research Group for Marine Geochemistry (Oldenburg, Germany). Samples were thawed, acidified with HCl (p.a. quality) to pH = 2 and purged with ultrapure synthetic air to remove inorganic carbon. DOC and TDN were measured with a total organic carbon analyzer (TOC-VCPH, Shimadzu) equipped with a TNM-1 module for nitrogen analyses and an ASI-V auto-sampler. A subset of samples was also filtered through pre-combusted Whatman GF/F filters. Because there was no detectable difference between filtered and non-filtered samples, our analyses of total dissolved organic carbon and nitrogen represents DOC and TDN. To test for possible matrix effects of the high salt content, a standard addition experiment was performed by adding NH_4_Cl and potassium phthalate to a subset of samples. The added amounts of DOC and TDN could be reproduced with an external calibration, demonstrating that the Dead Sea water matrix did not affect our analyses. Additionally the catalysts of the TOC analyzer where optically controlled for signs of chlorine breakthrough. The analyses of DOC and TDN were validated with consensus deep-sea reference material (CRM Program, http://yyy.rsmas.miami.edu/groups/biogeochem/CRM.html) provided by the University of Miami.

### Rare Earth Elements and Yttrium (REY)

REY were measured using ICP-MS (Elan-DRC, Perkin Elmer, Germany) as previously described [14]. Low concentrations of REY in the water made pre-concentration necessary. Approximately 3 L of filtrated water (0.22 µm, cellulose acetate Sartobran capsule filter, Sartorius, Germany) were spiked with a Tm-spike for recovery analysis and adjusted to pH 2 by using sub-boiled suprapure HCl (HCl_sp_ Merck, Germany) and within 2 h passed through a pre-conditioned C18 Sep-Pak cartridge (waters, USA) at a rate of 1 L h^−1^. Cartridges were pre-conditioned in the laboratory by cleaning them with 10 mL of 6 M sub-boiled HCl_sp_, rinsing in ultra-pure water (Merck Millipore, Germany) to neutral pH and loading with a liquid ion exchanger (a mixture of two different ethylhexylphosphates, Merck, Germany). The REY-loaded cartridges were washed with 50 mL of 0.01 M sub-boiled HCl_sp_ and eluted by 40 mL of 6 M sub-boiled HCl_sp_ at a rate of 3 mL min^−1^. The eluates were evaporated to incipient dryness and the residues dissolved in 3 mL of sub-boiled suprapure HNO_3_ (Merck, Germany) and spiked with a Ru-Re mixture that was later used to correct the internal drift of the response factors in the ICP-MS measurements, if necessary. The interference of molecular ions with the desired mono-charged ions of the REY were routinely corrected as previously described [12]. REY values are given in Table S1.

### DNA Extraction

Based on preliminary cell counts, 4 L of water and 25 g of spring sediments were used for DNA extraction as previously described [15]. All samples were incubated for 30 min in lysis buffer (0.1 M Tris, 0.05 M EDTA, 100 mM NaCl, 1% SDS, pH 8) at 100°C in a dry heating block or a water bath depending on sample size. Phenol was added (half of total volume) and samples were incubated at 60°C for 15 min. An identical volume of chloroform was added and, following 10 min of incubation at room temperature (RT) and 10 min of centrifugation, the aqueous phase was transferred. After a second chloroform extraction the DNA was precipitated overnight at **−**20°C with 1 volume of isopropanol and 0.05 volume of 5 M Sodium Acetate (pH 5.0). After a 20 min centrifugation the pellet was washed in 75% ethanol, recollected by centrifugation, and dissolved in 50 µL of molecular grade water. Due to the high salt content the DNA from the sediment samples was desalted using the Qiaex II gel extraction kit (Cat: 20021, Qiagen). DNA was quantified using a Nanodrop (Thermo Scientific) and subsamples of equal concentration were used for further analysis.

### Pyrosequencing

DNA extracts from a total of 18 samples were analyzed by 454 pyrosequencing for Bacterial and Archaeal diversity. Primer sets 28F and 519R [16] were used for Bacterial sequences and 341F [17] and 958R [18] for Archaeal sequences. Pyrosequencing was done by Research and Testing Laboratories, Lubbock, Texas, using a Roche 454 FLX Genome Sequencer system. Shortly, Tag-encoded FLX amplicon pyrosequencing (bTEFAP) was carried out as previously described by Dowd et al. [19]. A 20 ng (1 µl) aliquot of each DNA sample was used for a 25 µl PCR reaction. A 30 cycle PCR using HotStarTaq Plus Master Mix Kit (Qiagen, USA) were used under the following conditions: 94°C for 3 minutes, followed by 30 cycles of 94°C for 30 seconds; 55°C for 40 seconds and 72°C for 1 minute; and a final elongation step at 72°C for 5 minutes. Following PCR, all amplicon products from different samples were mixed in equal volumes and purified using Agencourt Ampure beads (Agencourt Bioscience Corporation, USA).

### Sequence Analysis

Diversity and community structure analyses were performed on 90,320 Bacterial and 41,111 Archaeal sequences obtained from samples of spring waters and sediments, biofilms and reference sediment. Sequence reads from PCR amplicon pyrosequencing were preprocessed (aligned and quality-controlled) by the bioinformatics pipeline of the SILVA rRNA gene database project [20]. Briefly, all reads were aligned using the SILVA Incremental Aligner against the SILVA SSU rRNA seed [20]. Non-aligned reads (putative contaminations/artifacts) have not been considered for further downstream analysis. Additionally, all remaining reads shorter than 50 aligned nucleotides and reads with more than 2% of ambiguities or 2% of homopolymers, respectively, were removed. Subsequently, reads of the filtered datasets were dereplicated, clustered and classified in parallel on a sample by sample basis. Dereplication (identification of identical reads ignoring overhangs) and clustering (OTU definition based on a non-redundant subset of reads) was done using cd-hit-est (http://www.bioinformatics.org/cd-hit) applying identity criteria of 1.00 and 0.98, respectively, both times with a wordsize of 8. For each OTU/cluster, the longest read was then used as a reference of this cluster for taxonomic classification. The classification was performed by a local nucleotide BLAST search against the non-redundant version of the SILVA SSURef dataset (release 106; http://www.arb-silva.de) using blast-2.2.22+ http://blast.ncbi.nlm.nih.gov/Blast.cgi) with standard settings. To filter out low identity and artificial BLAST hits, hits for which the function ‘(%sequence identity +%alignment coverage)/2’ did not exceed the value of 93.0 were discarded. For the analyzed reads with sufficiently good BLAST hits, the taxonomic classification of the best BLAST hit according to the SILVA taxonomy has been assigned to the read. Reads without any BLAST hits, or reads with weak BLAST hits only, were classified as ‘No Relatives’. Finally, the taxonomic path of each cluster reference was mapped to all reads within the corresponding cluster as well as to their corresponding replicates. This last step allowed to obtain quantitative information (number of individual reads representing a taxonomic path), within the bounds of PCR and pyrosequencing biases. To confirm the taxonomic affiliation of the sequences, all cluster references were imported into ARB [21] and inserted into the guide tree of the SILVA SSURef dataset (release 108).

A detailed summary of the 16S rRNA gene pyrosequencing data analysis process for each sample, including the total number of reads and length distribution, as well as the results of quality management, dereplication, and clustering, can be found in Table S2. A detailed list of the final taxonomic affiliation of all analyzed sequences together with their relative abundances within the amplicon pool are given in Tables S3 (Bacteria) and S4 (Archaea).

The sequences were deposited at the Sequences Read Archive (SRA) under study accession number ERA116549.

### Rarefaction Analysis

Rarefaction curves [22] were calculated for each sample. For each curve 100 data points were calculated choosing random sub sets of classified reads from the sample. Reads excluded by the aligner or the quality control, were not considered for the calculation of rarefaction curves. The first calculated data point always used a sample size of 1, while the last data point included all reads from the sample. The simulated sample sizes for the remaining 98 data points were evenly distributed between 1 and the size of the sample.

### Automated Ribosomal Intergenic Spacer Analysis (ARISA)

ARISA was done as previously described [23] using 3 replicates for each DNA extract. Several sediment samples from each spring were used as biological replicates. No biological replicates are available for the water samples.

### Fluorescence in Situ Hybridization (FISH)

Samples for FISH were fixed within minutes of sampling termination using fresh formaldehyde (1% final concentration), and stored at 4°C. Several replicates of 100 mL were filtered from each spring. Prior to permeabilization filters were embedded in low-gelling-point agarose (0.2% [wt/vol] in Milli-Q water), dried for 15 minutes at 37°C and dehydrated in 95% (vol/vol) ethanol. Subsequently, filter pieces were incubated in lysozyme solution (10 mg/ml lysozyme in 0.1 M Tris-HCL [pH 7.4] and 0.05 M EDTA [pH 8]) for 1 hour at 37°C. After two washing steps in Milli-Q water, a second permeabilization step was carried out using achromopeptidase (30 U mL^−1^ achromopeptidase in 0.01 M NaCl and 0.05 Tris-HCl [pH 7.4]) for 30 minutes at 37°C. For inactivation of intracellular peroxidases, filters were incubated for 30 minutes at RT in methanol containing 0.15% H_2_O_2_, washed in Milli-Q water, dehydrated for 1 minute in 95% (vol/vol) ethanol, and air-dried at room temperature. Filters were cut in sections and hybridized with the oligonucleotide probes EUB338 I–III [24], Arch915a and NON338 [25]. Formamide concentration in the hybridization buffers was 35% (vol/vol) for all probes used.

Hybridization and amplification was performed as described by Tujula et al. [26], with the following modifications. Hybridization was performed overnight (12–15 h) at 46°C. Amplification was increased by adding 3 parts of Alexa 488 (1 mg mL^−1^) labeled tyramides to 1000 parts of amplification buffer. The amplification time was elongated to 30 minutes at 46°C. The filters were counter-stained and mounted using a DAPI (4′,6-diamidino−2-phenylindole) mix as described by Teira et al. [27].

The samples were examined with a Zeiss Axioplan microscope with a 100-W Hg lamp and filter sets for DAPI and Alexa488. From each filter 5 to 10 different fields of view (around 1000 to 5000 cells) were enumerated using the ACME software by Michael Zeder (http://www.technobiology.ch).

### Hyper-spectral Imaging

High spatial resolution distributions of pigments in green biofilms covering cobble from spring 12 were measured using hyper-spectral imaging [28]. This method provides full spectral data per pixel of acquired image thus enabling a non-intrusive study of pigment distribution across the sample. Undisturbed biofilm samples were illuminated with a halogen lamp emitting in visible and near-infrared regions (400–1000 nm), and scanned with a hyper-spectral camera (Pika II, Resonon) from a distance of ∼5 cm at velocity 200 µm s^−1^. Subsequently, a biofilm subsample was placed on a microscope slide and scanned in a transmission mode under a Zeiss Axiophot microscope at velocity 2 µm s^−1^. Pigments were identified based on their *in vivo* absorption maxima (676 nm for chlorophyll *a*, 625 nm for phycocyanin, and 740 nm for bacteriochlorophyll *c*), and localized by calculating in every pixel of the image the second derivative of the spectral reflectance at the corresponding maximal absorption [28].

### Cluster Analysis

The different samples of the water and sediment microbial communities were compared by cluster analysis. The clustering was done using the DICE algorithm as implemented in the PAST software [29], using stress factors 13% and 7% for the 454 data and ARISA data, respectively.

## Results

### Water Chemistry

Compared to the Dead Sea water (data taken from Möller et al. [30]), underwater springs were significantly less saline and had a higher pH (Table 2). The southern springs were more saline and had a lower pH than the northern ones, with the exception of spring 1A, which was inactive at the beginning of the campaign and was sampled after it became active. All underwater springs except spring 2 had negative redox potential. Sulfide was detected in springs from both systems and ranged between 50–130 µM. DIC concentrations in the shore and underwater springs were 3–5 times higher than in the Dead Sea. DOC and TDN concentrations in underwater and shore springs as well as in the mixture of waters from the underwater spring 5 and the Dead Sea were also higher, however the DOC:TDN ratios were about 4 times lower than in the Dead Sea (Table 2).

**Table 2:** Water chemistry of underwater springs and auxiliary sites.

			Na^+^	K^+^	Mg^2+^	Ca^2+^	Cl^−^	SO_4_^2–^	Br^−^	TDS	Density	pH	Eh	H_2_S	DIC	TA	DOC	TDN	DOC/TDN	NH_4_^+^	NO_3_^−^	PO4_3_^−^	Mixing (%)	
			mM	mM	mM	mM	mM	mM	mM	g/L	g/cm^3^		mV	mM	mM	mM	µM	µM		µM	µM	µM	PW/Q	
		**Dead Sea**	1460	201	1952	508	6147	1.39	81.3	338	1.240	6.16	293	**−**	1.05*	4.1–4.7†	185	20	9.2	600#	3–8#	0.2#		
	**L**	**Spring 1**	42.9	5.23	34.8	12.6	127	5.00	5.10	7.58	1.003	7.44	–9	–	3.56	2.84	–	**−**		**−**	**−**	**−**	2.0/−	−/4.0
	**D_1_**	**Spring 1A**	464	56.7	502	137	1693	25.5	18.0	88.4	1.069	6.75	-86	-	3.40	3.44	-	-		**−**	**−**	**−**	25.3/−	
**N.**	**D_2_**	**Spring 2**	102	9.86	62.1	21.3	259	6.73	2.29	9.40	1.005	7.38	+38	0.127	4.15	3.98	728	77	9.4	78	0.8	0.25	−/8.0	
	**L**	**Spring 3**	62.6	6.67	41.1	12.9	164	3.20	1.49	16.8	1.010	7.43	–83	0.127	5.27	5.1	213	24	8.9	20	4.5	0.42	2.7/−	−/5.2
		**Spring 4**	37.0	5.00	29.0	8.00	108	2.00	1.00	6.07	1.002	**−**	**−**	**−**	**−**	**−**	**−**	**−**	**−**	**−**	**−**	**−**	1.9/−	−/3.6
**S.**	**D_1_**	**Spring 10**	277	31.0	222	59.0	858	5.00	9.00	44.7	1.031	6.76	–38	0.048	**−**	**−**	518	158	3.2	131	1.47	0.81	13.3/−	
	**D_1_**	**Spring 11**	270	30.6	218	64.0	818	4.22	8.26	44.0	1.031	6.76	–40	0.063	**−**	**−**	473	170	2.7	181	1.01	0.69	12.9/−	
	**D_2_**	**Shore Spring 1**	29.5	3.72	22.7	7.91	80.5	1.03	0.70	4.96	**−**	7.17	52	**−**	4.67	4.28	729	323	2.3	**−**	**−**	**−**	1.4/−	−/2.6
	**L**	**Shore Spring 2**	30.8	4.23	22.9	7.72	84.7	1.20	0.74	5.20	**−**	7.22	128	**−**	4.97	4.6	1445	593	2.4	**−**	**−**	**−**	1.5/−	−/2.9
	**D_1_**	**Shore Spring 3**	103	4.36	30.9	11.9	166	1.67	0.84	10.4	**−**	7.16	–47	**−**	5.80	5.32	1386	584	2.4	**−**	**−**	**−**	2.1/−	−/4.2
		**Spring 5**	**−**	**−**	**−**	**−**	**−**	**−**	**−**	**−**	**−**	**−**	**−**	**−**	**−**	**−**	736	394	1.9	**−**	**−**	**−**		
		**Porewater (PW)**	1508	233	2132	508	6365	2.89	62.3	348	**−**	5.66	247	**−**	6.20	1.80	**−**	**−**		**−**	**−**	**−**		
		**Qedembrine (Q)**	1254	110	965	367	3532	9.3	26.2	198	**−**	6.31	–97	**−**	4.35	2.76	**−**	**−**		**−**	**−**	**−**		
		**Jericho 5 well (JGA)**	3.67	0.14	2.09	2.28	8.04	0.8	0.05	0.82	–	7.20	368	**−**	4.78	4.32	**−**	**−**		**−**	**−**	**−**		

* Taken from Stein et al. [93];

† Taken from Gross [47];

# Taken from Stiller and Nissenbaum [76].

Classification based on REY: L- Limestone group;D1- Dead Sea group, subgroup 1;D2- Dead Sea group, subgroup 2.

Chemical composition and other characteristics of the waters from the Dead Sea, underwater springs, shore springs and additional auxiliary sites. Mixing coefficients represent the calculated percentages of brine (either porewater or the Qedem brine) admixed to the freshwater from the Jericho 5 well that best explain the measured concentrations of K, Cl and Br as well as the ratios of Cl/Br and Ca/Mg. PW/− denotes mixture of the porewater and freshwater, −/Q denotes mixture of the Qedem brine and freshwater.

**Figure 3:**
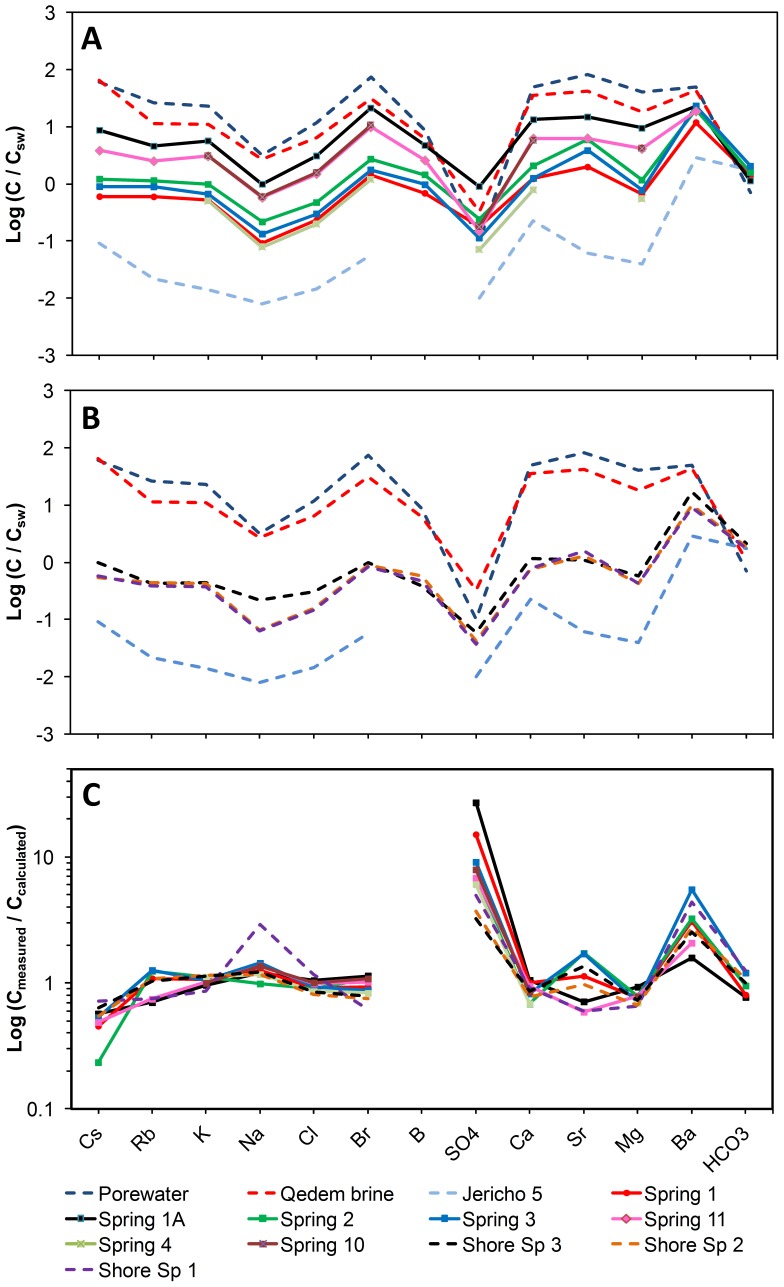
Seawater normalized (C_sw_) concentrations of major ions in waters from the underwater springs (A) and from reference sites (B). The concentrations are listed in Table 2 and in Table S1. The ions are arranged along the x-axis based on their natural behavior: heavy alkalis Cs and Rb are mainly controlled by surfaces such as those of clay minerals; K, Na, Cl and Br stand for brines and salt minerals (halides); SO_4_, Ca, Sr, Mg, Ba and HCO_3_ represent dissolved species from carbonate-sulfate minerals (e.g., anhydrite/gypsum, aragonite and barite). All these minerals are abundant in the Dead Sea sediments. (C) Ratios between the measured ion concentrations and those calculated by a two-component mixing model (see Table 2 for the estimated mixing coefficients) using the Jericho 5 freshwater and either the Dead Sea pore water or the Qedem brine as end-members.

Concentrations of major ions (Table 2) and trace elements (Table S6) varied significantly amongst the underwater springs. Seawater-normalized concentrations (hereafter denoted by subscript SW) exhibited sub-parallel patterns that were similar for all underwater springs (Fig. 3A). These patterns resembled those found in the shore springs from the area as well as in locally occurring brines, such as the pore water from the exposed Dead Sea Group (DSG) sediment or the thermal brines from the Qedem area (Fig. 3B). Both spring waters and local brines had higher Br_sw_ and B_sw_ compared to Cl_sw_ and SO_4sw_, and depleted Na_sw_ and SO_4sw_. The chemical similarity of the spring waters and brines led to the assumption that spring waters emerging on the shore and underwater are the result of (i) mixing of brines with the fresh groundwater component available from the mountain aquifers and (ii) dissolution and precipitation of evaporates which are abundant in the surrounding of the Dead Sea.

To assess the contribution of mixing, we used a two-component mixing model to calculate the ionic composition of the spring waters. The local brine (either pore water or the Qedem brine) was used as the first component, whereas freshwater from the Lower JGA (represented by the Jericho 5 well) was used as the second component. The latter is justified based on the hydraulic studies of Laronne Ben-Itzhak and Gvirtzman [31], which modeled the flow from the buried Lower JGA to Darga, and of Möller et al. [30], which proved a considerable supply of freshwater from the northwest to the Darga region. The calculation revealed that springs 1A, 10 and 11 contained between 12.9% and 25.3% of pore water admixed to the JGA freshwater, whereas spring 2 was better explained as a mixture of 8% Qedem brine and JGA freshwater (Table 2). The composition of the less saline underwater springs 1, 3 and 4 and shore springs 1–3 was equally well explained by mixing the JGA freshwater with either 1.4−2.7% of pore water or 2.6−5.2% of the Qedem brine (Table 2).

These mixing coefficients could explain measured concentrations for most but not all components (Fig. 3C). For example, the measured concentrations of SO_4_ and Ba were clearly higher (4−25 fold for SO_4_, 1.5−5.5 fold for Ba) than the calculated ones in all springs, whereas the measured Sr content was slightly higher in springs 2 and 3 and lower in springs 1A and 11 than calculated.

**Figure 4:**
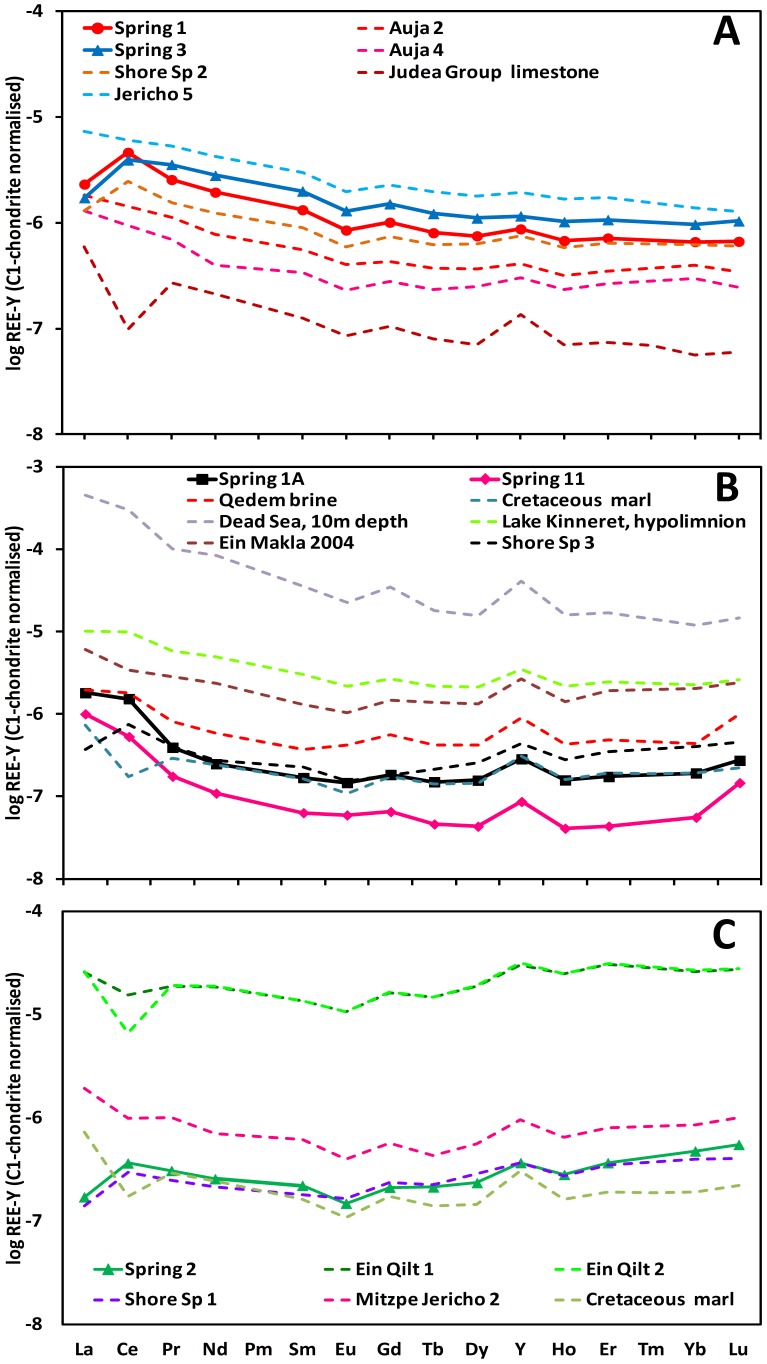
Rare Earth elements and Yttrium (REY) pattern in underwater springs (solid lines with symbols) and in diverse ground waters from the local area with comparable patterns (dashed lines). The origin of the different waters is explained in Table 1, the REY concentrations are given in Table S2. Whole-rock REY patterns for the Judea Group limestone and the Cretaceous marl are also presented (data taken from Möller et al. [28]). Their normalized values are shifted by 10^−7^ (limestone) and 10^−8^ (marl) to ease the comparison. The fractionation patterns separate the springs into two major groups, the “Limestone” group (A) and the “Dead Sea” group, which is divided into two subgroups (B–C). Note logarithmic scale in all panels.

The shore and underwater spring waters were characterized by distinct REY patterns, which allowed their classification into two groups (Fig. 4, Table 2, Table S1). In the “Limestone” group, which included underwater springs 1 and 3 and shore spring 2, the REY pattern continuously decreased from La to Lu, with positive Ce-, small positive Y- and small negative Eu-anomalies (Fig. 4A). These patterns resembled those found in waters from the Lower JGA (Auja and Jericho 5 wells) and in the whole Judea Group limestone [30], [32]. In the “Dead Sea” group, which included springs 1A, 11, 2 and shore springs 1 and 3, REY patterns had a patelliform shape. The subgroup that included springs 1A and 11 was characterized by significantly decreased medium REE resulting in a patelliform shaped pattern and positive Ce- and Y-anomalies (Fig. 4B), similar to the pattern found in the Qedem brine and the Cretaceous marl from the Judea Mountains [30], [32]. The second subgroup, which included spring 2 and shore spring 1 (Fig. 4C), was characterized by a gentler decrease from La to Gd and a stronger increase towards Lu. Such patterns are typical for waters coming from marly sequences of the Upper JGA, as represented by the Mitzpe Jericho 2 and Qilt springs, and similar to the whole rock composition of a Cretaceous marl from the Judea Mountains.

### Biofilms

Dense white biofilms covered sediments around the underwater springs at all sites. The biofilms around the northern springs 1–5 formed small thin patches adjacent to the water outlet (Fig. S2A). In contrast, biofilms around the southern springs covered relatively large (2–10 m^2^) patches of sediment next to areas where water seeped out without clear boundaries (Fig. S2B). Large areas covered with biofilms were also found on sediments with no detectable water seepage, such as on slopes below springs 10 and 11 at depth ∼20 m (Fig. S2C). Thickest white biofilms covering an area of several square meters were found around spring 12 (Fig. S2B). Microscopic analysis revealed that large, sulfur-storing filamentous bacteria, which are typical for sulfidic environments, were not present in the white biofilms.

**Figure 5:**
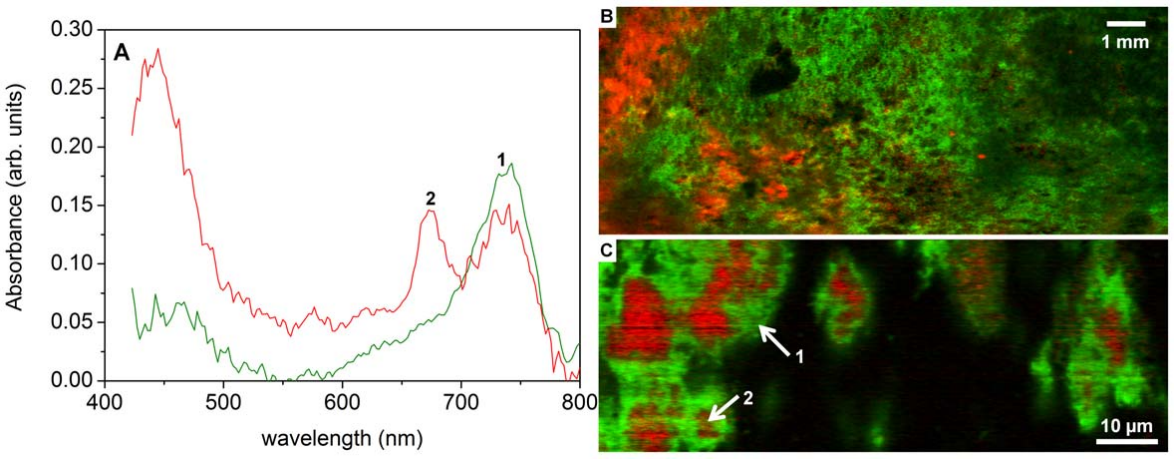
(A) Examples of absorption spectra of green biofilm samples from spring 12. Locations where these spectra were taken are shown by arrows in panel C. Major peaks at 675 nm and 740 nm correspond to in vivo absorption maxima of chlorophyll a and bacteriochlorophyll c, respectively. (B–C) Distributions of pigments in whole-biofilm samples (B) and inbiofilm samples under the microscope(C). Pigments characteristic for cyanobacteria (chlorophyll a and phycocyanin) are shown in red, whereas the pigment characteristic for green sulfur bacteria (bacteriochlorophyll c) is shown in green. Cyanobacteria were always co-localized with the green sulfur bacteria and never detected alone.

In addition to white biofilms, rocks around the southern springs (particularly around spring 12) were covered by thick green biofilms. The white and green biofilms covered exclusively the lower and upper sides of the rocks, respectively. Microscopic observations of the green biofilms revealed the presence of diatoms and unicellular cyanobacteria (Fig. S3). The latter was confirmed by hyper-spectral imaging, which revealed high concentrations of chlorophyll *a* and phycocyanin, characteristic pigments of this functional group (Fig. 5A). Hyper-spectral imaging additionally revealed a high abundance of bacteriochlorophyll *c*, a pigment characteristic for green sulfur bacteria, which was tightly associated with chlorophyll *a* (Fig. 5B). Microscopic observations showed that this association had a specific spatial structure, with patches of cyanobacteria surrounded by or co-localized with green sulfur bacteria (Fig. 5C).

### Microbial Community Analysis

**Figure 6:**
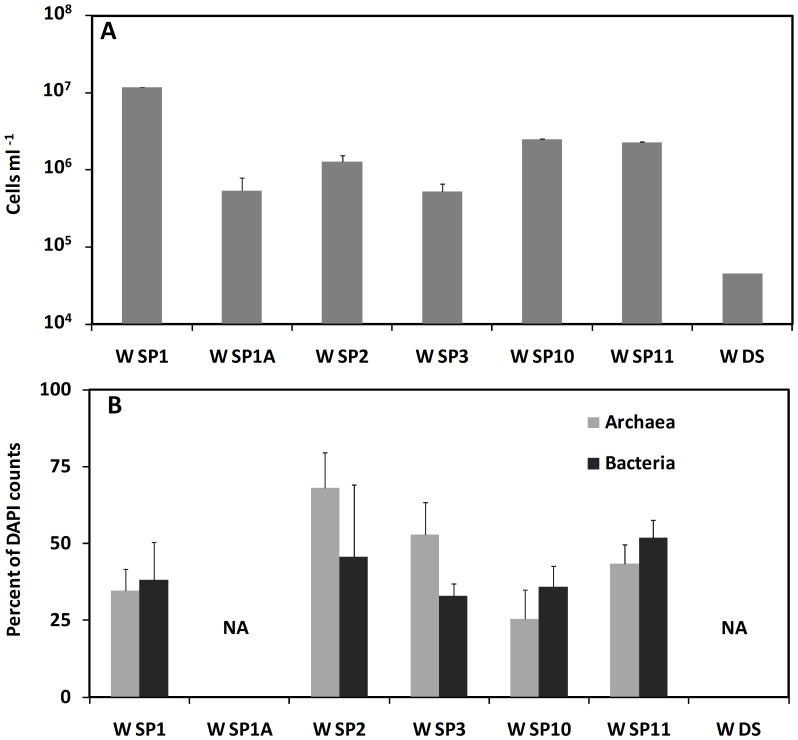
(A) Total counts of DAPI-stained cells and (B) percentage abundance of Archaea and Bacteria within the total cell counts in water samples collected from different underwater springs and from the reference Dead Sea water. Wn denotes water sample from spring n. Error-bars indicate standard error (N = 10); NA = data not available.

Cell densities in the spring waters ranged between 7×10^5^ and 10^7^ cells mL^−1^, and were between 10 to 100 times higher than in the ambient Dead Sea water (Fig. 6A). Bacteria made 30–50% of the total cell counts (Fig. 6B), whereas in ambient Dead Sea water where bacteria could not be detected [33]. Cell densities in sediment and biofilm samples could not be quantified in this study due to technical complications.

Rarefaction curves showed that the species richness was largest in the spring water samples (with the exception of spring 1A and 3) and progressively decreased in samples from the green biofilms, spring sediments and white biofilms (Fig. S4). Diversity in the spring sediments was similar to that in the reference DSG sediment.

**Figure 7:**
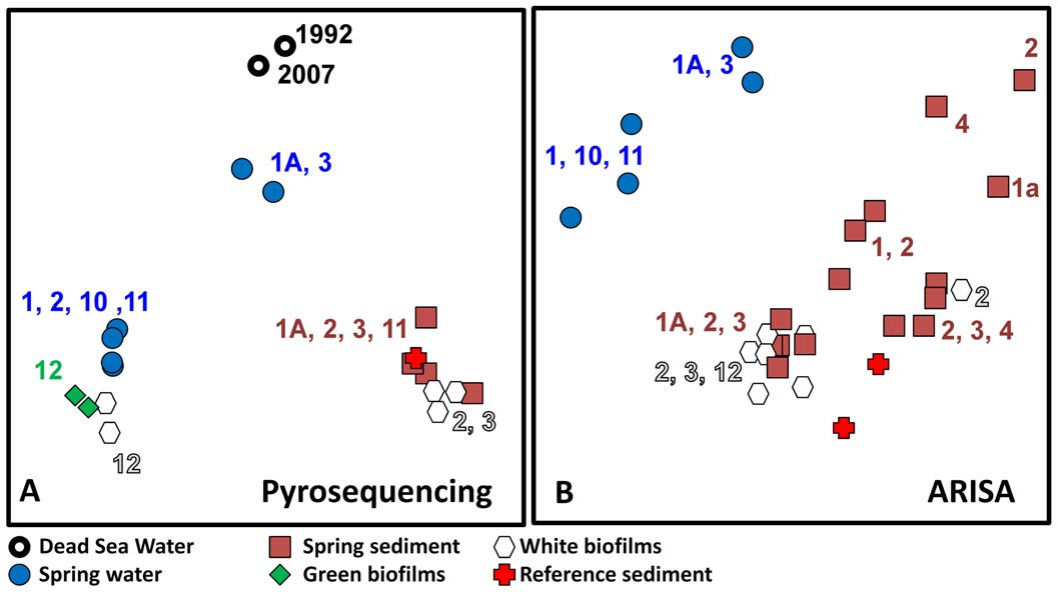
Non Metric Multidimensional Scaling (NMDS) plots derived by the DICE algorithm from the (A) 454 pyrosequencing and (B) ARISA data, using stress values of 13% and 7%, respectively. Clustering of the pyrosequencing data was performed on the data matrix produced by the NGS system at a taxonomic depth of 5 (Family level). Duplicate samples represent biological replicates. Data for the 1992 and 2007 analyses where obtained from Rhodes et al [31] and Bodaker et al [30] respectively.

Both pyrosequencing and ARISA analyses showed clearly that the microbial communities in the spring waters and spring sediments are different (Fig. 7; Table S5). Furthermore, the communities from the reference DSG sediment were much more similar to those in the spring sediments than those in the spring waters. The different replicate sediment samples used for the ARISA analysis showed no spring-specific clustering. The microbial communities in the green and white biofilms are closer to the spring water. Thus, the communities in the spring water and the biofilms differ from the Dead Sea communities but the sediments near the springs are colonized largely by normal Dead Sea microbial communities.

Based on species composition, with the exception of spring 11, the sediment samples share 45–50% similarity among themselves. A similar trend is observed among the biofilm samples; however, the sediment, biofilm and water sample clusters samples, were only 10% similar. When sequence frequency was additionally taken into account these values changed to 50–70% vs. 5% respectively (Fig. S5). All spring-associated communities were very different (maximum 10% similarity based on species composition) from the residual Dead Sea communities described by Bodaker et al**.** [33] as well as from the communities identified during the 1992 bloom linked to the dilution of the upper water layer of the Dead Sea [34] (Fig. S5). When comparing spring waters only, the communities in springs 1A and 3 stood out in both analyses. This difference was, however, more pronounced in the pyrosequencing data, presumably due to a lower number of sequences obtained from these samples. Pyrosequencing indicated that the microbial communities in the white biofilms covering cobble around spring 12 were much more similar to the water-borne communities from springs 1, 2, 10 and 11 than to the white biofilm communities from springs 2 and 3. This similarity was, however, not evident in the ARISA data.

**Figure 8:**
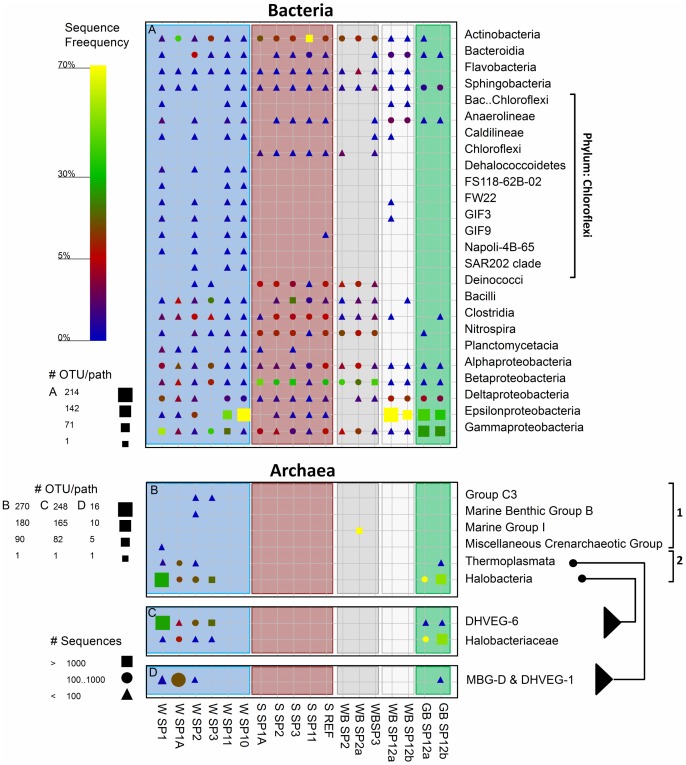
Graphical representation of the sequence frequency in the studied Dead Sea samples, showing major detected classes within the Bacterial and Archaeal domains. Classes belonging to Crenarchaea and Euryarchaea are marked by brackets 1 and 2, respectively. The Halobacteria and Thermoplasmata classes are shown also at the family level to facilitate a more specific sample comparison. The color of the symbol represents the relative frequency of the taxonomic path within the sample. The size of the symbol represents the number of OTUs at deeper phylogenetic levels within that taxonomic path (see Methods for the definition of OTU). The shape of the symbol represents the number of sequences in the specific taxonomic path. Columns are shaded according to the sample type: blue = spring water, brown = spring sediment, grey = white biofilms from northern springs, white = white biofilms from southern springs, green = green biofilms from southern springs. Abbreviations in sample names: W = spring water. S = spring sediment, WB = white biofilm, GB = green biofilm, S-REF = reference sediment from the Dead Sea.

The major classes of detected Bacterial sequences varied between the samples from the springs’ water phase, spring sediment and biofilm samples (Fig. 8A). Hereafter only the taxonomic name will be used when referring to sequence data. In spring water samples, *Epsilon*-, *Gamma*- and *Deltaproteobacteria* were detected in highest numbers, with *Epsilonproteobacteria* being especially dominant in southern springs 10 and 11, where they made up 75% and 47% of the total number of sequences, respectively. With the exception of springs 1A and 3, the spring water samples exhibited a large diversity within the *Chloroflexi* phylum. This was in contrast to sediment samples, where many classes from this phylum were absent, except for the *Chloroflexi* class itself, which was found exclusively in spring sediments. Another major difference was that the sediment samples contained many more sequences of *Deinococci*, *Clostridia*, *Nitrospira, Betaproteobacteria* and *Actinobacteria.* Except for *Clostridia*, these classes were frequent also in white biofilms from the northern springs 2 and 3. Sequences detected in the white biofilms from the southern springs were very different from those in the northern springs. They were dominated by *Epsilon*- and *Deltaproteobacteria* (on average 73% and 10%, respectively) and contained *Bacteroidia* (1.9%) and *Anaerolineae* (2.9%), unlike in other samples where these groups were not detected. Similar classes were detected also in the green biofilms from these springs, with *Gammaproteobacteria* and *Sphingobacteria* being additionally relatively abundant (25% and 1.5% on average, respectively).

Archaeal sequences were amplified successfully from only 11 out of the 18 samples. They contained a significant number of Bacterial false-positives (Table S4), which were excluded from the final analysis. Compared to Bacteria, the diversity of Archaea detected in the spring-associated samples was much lower, though in many samples (especially in spring sediments) no Archea were detected (Fig. 8B). *Crenarchaea* were detected in significant amounts only in one sample and clustered with sequences of uncultured deep subsurface *Crenarchaea* [35] within the Marine Group 1 *Crenarchaea.* The *Euryarchaea* comprised mainly *Halobacteria* and *Thermoplasmata*. Both of these groups clustered with sequences found in deep-sea hydrothermal vents (Fig. 8C), and their possible significance will be discussed later. The *Euryarcheal* community from the more saline spring 1A differed from the communities found in the other northern springs. While *Euryarchea* were detected only in water samples from the northern springs, in southern springs they were detected only in the green biofilms.

**Figure 9:**
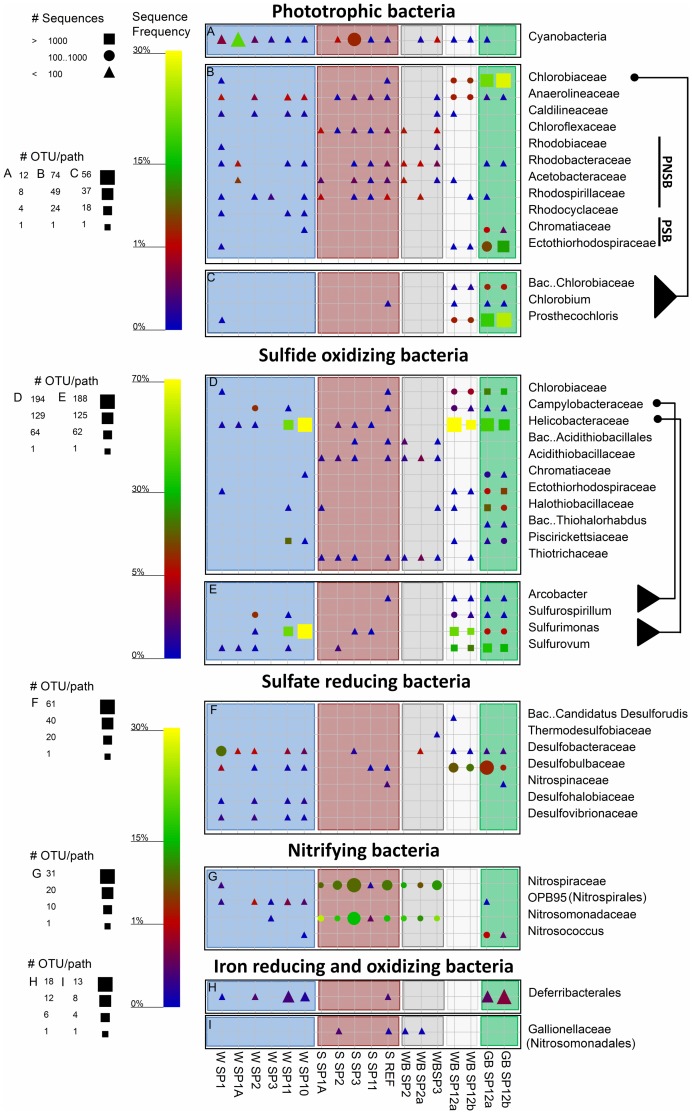
Graphical representation of the sequence frequency in the studied Dead Sea samples, showing major detected phyla and families of different functional groups of Bacteria. PSB and PNSB in panel B refer to purple sulfur and non sulfur bacteria, respectively. The different genera within the families Chlorobiaceae,Campylobacteraceae and Helicobacteraceaeare shown to facilitate a more specific sample comparison. The symbols and sample naming are explained in detail in Fig. 8. Note different legends for OTU/path for each panel, and scale-bars for relative sequence frequency for several combined panels.

The studied samples differed also in functional groups of Bacteria (Fig. 9). Phototrophic bacteria were detected in large numbers in green biofilms from spring 12 and in lower amounts elsewhere. Cyanobacterial sequences were detected in most samples usually making up less than 1% of the sequences. Only water from spring 1A and the sediment of spring 3 comprised 20% and 3.6% cyanobacterial sequences respectively (Fig. 9A). Although cyanobacterial pigments, as detected by hyper-spectral imaging, were often detected in the green biofilms of spring 12, only few cyanobacterial sequences were obtained from these samples. Attempts to use different sets of cyanobacteria-specific primers to identify these organisms resulted always in non-related sequences (*Halanerobiales*). Most abundant phototrophs in the green biofilms were green sulfur bacteria (25%), consistent with the presence of bacteriochlorophyll *c* (Fig. 6), and purple sulfur bacteria (10%) (Fig. 9B–C). Purple non-sulfur bacteria and *Chloroflexaceae* were common in sediment samples, but not in biofilms and spring water.

Chemolithotrophic sulfide oxidizers from the class *Epsilonproteobacteria* were very frequent and diverse in the green and white biofilms from spring 12 (on average 37% and 72%, respectively; Fig. 9D). The high abundance of this group was shown also by FISH analysis (Fig. S6). The water of the nearby springs 10 and 11 contained *Epsilonproteobacteria* as well; however, only from the *Sulfurimonas* genus and almost no sequences of *Sulfurovum* or the *Campylobacteraceae* (Fig. 9E). Few (<3%) *Acidithiobacillaceae* and *Thiotrichaceae* (recently renamed as *Beggiatoaceae* [36]) were found around most springs, except around spring 11.

Sulfate reducers were found at relatively higher numbers mainly in the water samples and in the biofilms from spring 12 (Fig. 9F). Within this group, *Desulfobacteraceae* were dominant in the water samples (0.4–8%) while *Desulfobulbaceae* were dominant in the biofilms (2.7–9%). The sulfate reducers in the spring water were more diverse but 4–8 times less frequent than in the biofilms associated with spring 12.

Nitrifying bacteria were highly abundant in the spring sediments from the northern system (11–27%) and in lower amounts in spring waters (<2%; Fig. 9G). The water samples contained mainly an unnamed family of *Nitrospirales* (OPB95), whereas *Nitrospiraceae* and *Nitrosomonadaceae* were abundant in the sediment samples and white biofilms around springs 2 and 3. *Nitrosococcus* were identified in the green biofilms of spring 12 (0.5–1.2%). Remarkably, nitrifiers were found with an equally high frequency in the Dead Sea reference sample as well. The ammonia oxidizing bacteria from the family *Nitrospinaceae* (*Deltaproteobacteria*) were found exclusively in the reference sediments (0.4%; not shown in Fig. 9G).

*Deferribacterales* (iron and nitrate reducing bacteria) were found in the green biofilms of spring 12 (0.6%), in the reference sediments (0.4%), and in all spring water samples (<0.41%) except for springs 1A and 3 (Fig. 9H). A low amount of sequences of iron oxidizing bacteria belonging to the *Gallionellaceae* (<0.1%) was detected in sediments and white biofilms around spring 2 and in the Dead Sea sediment (Fig. 9).

## Discussion

This study provides the first description of dense microbial communities in the Dead Sea. It shows that these communities are exclusively linked to groundwater seepage at the lake floor. Here we discuss the possible source of these organisms and the putative biogeochemical pathways they are mediating. Furthermore we discuss the origin and chemical properties of the spring waters and their importance for the microbial communities.

### Origin and Properties of the Underwater Springs

The major elements composition as well as the rare earth elements and yttrium (REY) patterns are markers of biotic and abiotic processes that occur along the flowpath of the groundwaters before they emerge as springs at the Dead Sea floor. We combine these data with the results of the bacterial community analysis to identify these processes.

Generally, in groundwater systems, REY are mainly released from accessory minerals, among which phosphates and carbonates predominate [37]. After dissolution of REY-bearing minerals, the majority becomes immediately adsorbed onto mineral surfaces [38]. Consequently, along the flow path, mineral surfaces in contact with passing water are continuously equilibrated to the concentration of REY in the water. Once a hydrological system is well established, changes in REY no longer occur or they are at least insignificant. Hence, REY patterns of such waters represent the initial water-rock interaction in the recharge area of the aquifer that defines the primary REY pattern of the groundwater [30], [32].

The REY patterns of the studied springs are evidence of freshwater running from the Judea Group Aquifer (JGA) to the Dead Sea. However, they show distinct features from which their contact with the DSG sediment can be inferred. Waters from the “Limestone” group springs show similar REY pattern as freshwaters pumped from the Lower JGA (wells Auja 2 and 4, Jericho 5). We therefore assume that these waters flow from the limestone aquifer to the Dead Sea through well-developed open cracks with negligible contact to the unconsolidated DSG sediments. By flushing trough these cracks and by admixing of interstitial brines, the waters are further enriched with dissolved and suspended minerals, as well as species such as H_2_S, but only in marginal amounts.

In contrast, we suggest that waters in the “Dead Sea” group springs migrate from the JGA through small fissures and less open cracks, with their flow paths through the DSG sediment possibly still developing. Consequently, their interaction with DSG minerals and interstitial brines is much more intense. This is supported by the similarity of their “soup-bowl”-like REY pattern to that of the Qedem brine, for which the intense contact with DSG sediments has been shown [39]. However, this similarity cannot be explained by simple admixing of the local brines to the JGA freshwater. If the mixing coefficients estimated from the major ion composition (Table 2) were used, the resulting REY patterns would be much closer to that of the JGA than those of the pore water or Qedem brines (Fig. S7). Therefore, additional processes need to be considered. First, the pattern with considerably decreased medium REE’s (MREE) and positive Y-anomalies can be the result of long-term leaching of phosphates and sulfates. This process releases predominantly MREE [37]; thus, if it occurred in the past, the MREE would now be lacking and thus lead to MREE depletion in the passing water. Second, the observed pattern can be due to FeOOH precipitation [40] and subsequent weathering. If FeOOH complexes at pH>6, a relevant value for these springs, Ce, Nd and Sm as well as heavy REE have a higher affinity to co-precipitate than the rest of the REE and Y [40]. Therefore, the remaining waters show depletion in medium REE and slight increase towards Lu with a positive Y-anomaly. However, if FeOOH become reduced, heavy REE and Ce are predominantly released and become enriched in the water [26]. Indeed, spring waters 1A and 11 are reducing (Table 2) and show patterns with positive Ce-anomalies, which are comparable to those in waters from the anoxic hypolimnion in Lake Kinneret and from strongly reducing Ein Makla spring in Hammat Gader (Fig. 4B), where redox-cycling of Fe plays an important role [32], [41]. Water of spring 2 interacts with FeOOH as well. However, in contrast to the other “Dead Sea” group members, recycling of Fe-oxihydroxides is unlikely in this spring due to its oxidizing conditions (Table 2). Instead, FeOOH precipitated along the flow path is weathered and not reduced, releasing heavy REE first and preventing Ce-anomalies, the latter because Ce is still in an oxidized state (Ce(IV)) and therefore more stably fixed in the Fe complexes [42]. Hence the REY pattern for spring 2 shows a less decreasing trend from La to Eu than the pattern of springs 1A and 11 (compare Figs. 4B and 4C). The initial source of iron, which is necessary for complexation of FeOOH, may be the weathering of Fe-bearing cherts. These are abundant in the Senonian rocks of the Judea Mountains [43] and in the eroded wadi debris, therefore also in the DSG-sediments [44].

The ionic composition of the spring waters indicates that the JGA freshwaters are affected by mixing at different proportions with the locally occurring brines (either the Dead Sea pore water or the ascending Qedem brine) before they emerge at the Dead Sea floor. The estimated pore water content in the more saline springs 1A, 10 and 11 was relatively high (13–25%). The less saline springs 1–4 could be equally well explained by mixing of JGA water with either 1.4–4.1% of pore water or with 2.6–8.0% of Qedem brine. This uncertainty was due to the relative similarity of the two brines with respect to their major ion composition. However, it could be resolved by considering molar Mg/Ca-ratios, which were quite distinct for the two brines (4.11 in the pore water, 2.6 in the Qedem brines). Since spring waters mainly comprise JGA freshwater with molar Mg/Ca<1, their high molar Mg/Ca ratios (2.76 to 3.76), which are higher than that for the Qedem brine, must be the result of a significant Mg-source. This could be either the admixed pore water or dissolved Mg-rich minerals. Such minerals (e.g. carnallite, bischofite) have been described for sinkholes [45] and most probably exist also within the thin DSG-sediment crust [46],where evaporation lead to the precipitation of such minerals However, they cannot be found in depths where the groundwater emerging in the studied springs is assumed to flow. Hence, the high molar Mg/Ca ratios in the spring waters indicate a significant contribution of the Mg-rich pore water as the preferential mixing partner, but do not exclude the contribution of Qedem brines as a possible third component.

The ionic composition additionally indicates that the groundwaters are affected by interactions with clays and by dissolution of evaporates such as aragonite, gypsum, anhydrite and halite, all of which are present in DSG-sediments [47]. Since Cs and Rb are highly controlled by surfaces, intense clay-water interaction is suggested by the marked differences between the measured Cs and Rb concentrations and those calculated by the mixing model (Fig. 3C). The moderately high DIC in the spring waters is predominantly in the form of HCO_3_, suggesting dissolution of calcite (in the JGA) and aragonite (in the DSG sediment). The HCO_3_ content in the brines stays stable suggesting the high DIC (up to 6.2 mmol L^-1^) is not a result of additional mineral dissolution. Instead, the higher DIC concentration is due to increased CO_2_, originating probably from bacterial decomposition of organic matter in the DSG-sediment.

The DOC in the underwater springs is high. This is probably due to mixing of the spring water with the pore water (see above), which is rich in organics (Siebert, unpublished data) due to continuous burial of terrestrial organic material during the sedimentation process of the Dead Sea sediments [44]. High DOC was also detectable in the buoyancy-forced flow of underwater spring 5 as sampled on the surface. This suggests the underwater springs are a source of organic matter input into the Dead Sea. The low DOC:TDN ratios (<3) in the majority of the springs suggest that most of the dissolved nitrogen is inorganic [48]. The high concentration of ammonium is derived probably from the oxidation of organic matter during the process of sulfate reduction, as described for other subsurface aquifers in the Dead Sea area [49]. Although exhibiting the same major ions and REY pattenrs, the underwater springs in the northern system (2 and 3) have a higher DOC/TDN ratio compared to the other springs; however, as most of the N is accounted for as ammonium we suggest that a large part of it was consumed as a nutrient by the microbial community along the aquifer flow path through the DSG sediments.

In some underwater springs, H_2_S was detected in relatively high concentrations. This H_2_S possibly originates from bacterial sulfate reduction that occurs along the flow path of the spring water, as shown for other sulfidic spring systems [49], [50]. This process requires organics and dissolved SO_4_. The latter may be provided to the hydro-biological system by mineral dissolution, or by active bacterial leaching of anhydrite from the DSG sediment, as indicated by the high B_SW_/SO_4SW_ ratios. The dissolution of anhydrite and gypsum is possible, since spring waters are undersaturated with respect to these minerals (Table S6). Indeed, spring waters show elevated SO_4SW_ concentrations, which exceed those in the Dead Sea, the pore water and the JGA groundwater, but are lower than in the Qedem brine (except for spring 1A). Accompanying the dissolution of anhydrite (CaSO_4_) is the release of Ca. However, enrichment in Ca-ions in spring waters is prevented by simultaneous precipitation of aragonite, supported by the fact that, in their outlets, all spring waters are supersaturated with respect to calcite and aragonite (Table S6).

Underwater spring waters showed remarkably high Sr_SW_/Ca_SW_ ratios (>1). These can be explained by several processes, such as (i) dissolution of celestite (Table S6), which is found among the precipitated minerals in the Dead Sea [51], [52], (ii) dissolution of Sr-containing anhydrite [42], or (iii) release of Sr during transformation of aragonite to calcite [53].

In conclusion, the freshwater input in the brackish underwater springs originates from the Judea Group Aquifers (JGA). However, as suggested by our water chemistry data and argued in detail above, the JGA waters are affected by a number of processes before they emerge at the Dead Sea floor. These include interaction with clay minerals, fine clastics and FeOOH complexes, dissolution or precipitation of different types of evaporates (e.g., aragonite, gypsum, anhydrite, halite, celestite, barite), bacterial degradation of organic matter (by sulfate reduction), and mixing with interstitial and ascending brines in the Dead Sea Group sediment. The sum of these processes results in water with high concentrations of dissolved ammonium and organic matter, sulfide, sulfate and phosphorus; all of which are necessary to support the microbial communities at the springs outlets in the Dead Sea, as discussed below. Although the springs emerge over a relatively small area at the Dead Sea floor, our data indicate that their flow between the graben shoulders and the lake occurs through pathways of different hydraulic conductivities. This leads to various residence times, which in turn results in a variable extent to which the above processes affect the spring waters’ chemical composition. Overall, spring waters in the “Limestone” group (springs 1,3, shore spring 2, and likely also spring 4) probably flow through well-developed open cracks, allowing marginal water-mineral interaction. In contrast, waters in the “Dead Sea” group (springs 1A,2,11, shore springs 1 and 3, and likely also spring 10) are likely to flow from the JGA through smaller fissures and less open cracks, with their flow paths through the DSG sediment possibly still developing. Consequently, their interaction with minerals and interstitial brines is much more intense.

### Source of Microorganisms in Spring Waters

Microbial communities in the spring waters contained high cell densities. The close match between the total cell counts obtained by FISH and DAPI staining is a good indication that the majority of these cells were alive at the time of sampling [54], [55]. Although the microbial community in the Judea Group Aquifer has not been studied yet, pristine aquifers always have very low numbers of microbial cells [56]–[58]. Therefore, we suggest that these cells detected in the spring waters grew and were collected along the path of the groundwater flow from the aquifer to the Dead Sea and did not grow in the aquifer itself.

This conclusion is supported by the fact that the spring waters contain solutes that can be utilized or produced by microbial metabolisms. The sulfide in the spring waters probably originates from sulfate reduction, which is a feasible process as both sulfate and DOC are available. The high ammonium concentrations are probably the result of organic matter oxidation and are a further support for microbial activity along the aquifer’s flow path. The REY patterns further suggest that redox cycling of Fe occurs along the flow path of the “Dead Sea” group springs. The requirement for Fe reduction are met by the availability of DOC and FeOOH, while Fe oxidation may occur anaerobically [59], [60] or in parts of the aquifer that are still oxygenated. Oxidation of organic matter is further supported by the high concentration of inorganic nitrogen.

The source of microorganisms in the spring waters by collection along the path is further supported by the relatively high fraction of detected Archaea, which was much higher than in other groundwater aquifers and freshwater bodies [61], [62]. These Archaea may originate from two different possible sources. Shoreline pore water can be the first source as suggested by the fact that majority of the detected Archaeal sequences were from the halophilic group *Halobacteria*, which usually require a minimum salinity of 15%. The pore water of the Dead Sea shoreline is saturated with brine and contains organic matter from flood carried debris (C. Siebert, unpublished), which provides suitable conditions for *Halobacteria*. Furthermore, no *Halobacteria* were detected in springs far from the sandy shoreline, i.e. at the bottom of a steep cliff (springs 10 and 11).

An additional or alternative origin of the Archaea could be a second water source. This is suggested by the fact that the majority of *Halobacteria* detected in the spring water fall within a cluster of organisms associated with deep hydrothermal vents and differ from the *Halobacteraceae* found in the springs sediments (Fig. 8C). It has been shown before that this deep sea group of the *Halobacteria* can thrive in less saline environments [63], [64]. The *Thermoplasmata*-associated sequences from the springs cluster with samples from deep hydrothermal vents as well. The Dead Sea is located in a tectonically active environment [65] and ascending hydrothermal brines are often observed as thermal springs(e.g. Ein Gedi and Mitzpe Shalem) [41], [65], [66], [67]. From their REY patterns and composition of major ions it cannot be excluded that ascending hydrothermal brines, as observed in the Qedem area, are intruding into the waters of the studied spring system.

### Source of Microorganisms in Spring Sediments

Our data show that majority of organisms brought in by the springs do not colonize the sediments around them. Instead, the dense and diverse microbial communities found in the sediments around the springs consist largely of organisms that are resident in the Dead Sea sediments. This conclusion follows from a detailed comparison between the microbial communities detected in spring waters, spring sediments, Dead Sea water column [33] and the Dead Sea during a bloom [34]. First, microbial communities in spring sediments were much more similar to those found in the ambient Dead Sea sediment than to those detected in the spring waters The larger spread of sediment samples in the ARISA analysis could be a result of the higher number of samples, compared to the 454 analysis, as well as of the higher resolution of an ITS based method in comparison with partial sequencing of the 16S rRNA gene. The spread away from, rather than around, the Dead Sea reference sediments is probably due to an uneven number samples of Dead Sea reference sediments as compared to spring sediments Second, microbial communities associated with springs differed from those induced by freshwater input into the Dead Sea during surface floods. Bodaker et al. [33] showed that the bloom community of the Dead Sea differed from the residual community in the water column. Our data shows that these two communities resemble each other more than they resemble the communities associated with spring waters or sediments. Whether the dense microbial communities found associated with the springs on the lake’s bottom are a result of lowered salinity or an input of nutrients dissolved in the spring water needs to be further investigated.

### Putative Biogeochemical Pathways

The importance of a certain metabolic pathway is linked to the abundance and activity of the cells involved. Therefore, solid data on the actual abundance of cells (obtained by FISH), as well as direct evidence that the cells detected by FISH actually perform this metabolism *in situ*, is required. As we at present do not have all this information, we base the following discussion on the assumption that the importance of a given metabolism is approximated by the relative frequency of detected sequences that are closely related to species for which the metabolism in question was proven. Exceptions to this approach are phototrophy and sulfide oxidation, whose importance is further supported by our pigment analyses and visual *in-situ* observations of thick white biofilms, respectively. Overall, our complete dataset suggests that photosynthesis, sulfide oxidation, sulfate reduction, nitrification, and iron reduction most probably occur in the underwater spring ecosystems.

Phototrophy appears to be of significance only in the vicinity of spring 12, as thick green biofims were found only there, mostly adhered to cobble. Between 30–40% of the sequences obtained from these biofilms are of known phototrophs. Spring 12 is shallower (12–14 m below water level, on a slope), not located within a crater structure, and the water flows through pebbles and cobble, thus not creating clouds of fine sediments. This was probably the only site where the light intensity (up to 45 µmol photons m^−2^ s^−1^ in mid day) was sufficient to fuel a dense phototrophic community. The dominant phototrophs in these biofilms were green sulfur bacteria, which are known to adapt well to low light intensities [68]. Sequences of purple sulfur bacteria, mainly represented by the *Ectothiorhodospiraceae*, were also highly abundant, making up 6–12% of the sequences. Though we could identify cyanobacteria microscopically (based on specific absorption and autofluorescence of their pigments), we obtained only a few sequences of a *Chroococcidiopsis*-like organism.

Hyper-spectral imaging showed a peculiar association between the green sulfur bacteria and the cyanobacteria, with patches of cyanobacteria surrounded by or co-localized with green sulfur bacteria. A co-localization of cyanobacteria and green sulfur bacteria was not described before. Green sulfur bacteria are usually strictly anaerobic organisms, and contain a quenching mechanism for protection against oxidation [69]. On the other hand, although cyanobacteria that are able to switch to anoxygenic photosynthesis are also known [70], [71], cyanobacteria are oxygenic phototrophs.The nature of the association found in this study is unclear and will be the subject of future studies.

Sulfide oxidation appears to be the key metabolism in this ecosystem, as suggested by the presence of a large variety of sulfide oxidizing bacteria. In the biofilms from the southern system 75–85% of the sequences are associated with known sulfide oxidizing bacteria. In the northern system the percentage is much lower (1.5–4.5%). Hydrogen sulfide (H_2_S) was measured in significant concentrations in the underwater springs (Table 2) and could be often smelled from the freshly retrieved sediment cores and from shore springs. Oxygen is present in the Dead Sea waters (20–40 µM; [72]); thus the process of aerobic sulfide oxidation is feasible. The sulfide oxidizing communities in the southern springs are different from those in the northern springs. The southern springs consist mainly of *Epsilonproteobacteria* and phototrophic sulfide oxidizers, while the *Thiotrichaceae* and *Acidithiobacillaceae* inhabit sediments and biofilms around the northern springs. The difference in sequence abundance between these spring sites suggests that sulfide plays a more important role in the southern system. This is also evident by the significantly larger size of white biofilms in the southern springs. Interestingly, sulfide oxidizers were not found in the water of all springs. The large number of sequences obtained from the water of springs 10 and 11 suggests a thriving community of sulfide oxidizing bacteria along the path of the water flow. The genera of sulfide oxidizing *Epsilonproteobacteria* found in the water of springs 10 and 11 differ from those found in the biofilms of spring 12. This supports the hypothesis mentioned above that only a few of the organisms found in the spring-associated biofilms and sediments, originate from the spring water. *Epsilonproteobacteria* are common among the non-phototrophic sulfide oxidizing bacteria in moderate saline environments [73] and were found to be main players in salt gradient systems such as the deep sea Mediterranean brines [74], [75].

Sulfate reduction is probably the source of the sulfide both in the spring waters and in the biofilms. This is consistent with the presence of diverse groups of sulfate reducers detected in most water samples. In the green and white biofilms of spring 12 sequences of sulfate reducers make 4% and 11% of total sequences respectively. The latter was confirmed by FISH using a specific probe for *Deltaproteobacteria* (Fig. S2). Along the water flow path sulfate necessary for the process originates from gypsum and anhydrite dissolution. In the biofilms, the co-existence of sulfate reducers with sulfide oxidizers suggests that an internal sulfur cycle exists in these communities.

Sulfate reducers are generally missing from the sediments of the northern springs. Sulfate reduction was never convincingly measured in the Dead Sea sediments (Oren A, unpublished results), and to date no extreme halophilic sulfate reducers are known. The fluctuating water flow, observed in the northern system, may not allow halotolerant sulfate reducers to develop there, as the salinity is regularly too high. In turn, the lack of sulfate reducers, the fluctuating water flow and the sediment instability are probably the reasons for the low abundance of sulfide oxidizers in the northern springs.

Nitrification appears to be significant in the sediments and biofilms of the northern springs and is absent in the southern system. Nitrogen is not limiting in the Dead Sea or in the underwater springs (Table 2). Furthermore, up to 400 µM of ammonia were measured previously in the Dead Sea water column [76]. As nitrogen is plentiful, the reason for the absence of this group from the southern springs cannot be explained at this moment.

Reduction of iron, and possibly of other metals, may also be significant in the spring system. The *Acidithiobacillaceae* sequences (0.9–3%) from the northern springs are affiliated with the genus *Acidithiobacillus* and more specifically with *Acidithiobacillus thiooxidans* and *Acidithiobacillus caldus*, which are both known for dissolution of metal sulfides [77]–[79]. There are several other groups of organisms detected that suggest the role of metal reducing and oxidizing bacteria in the system. For example, members of the phylum *Deferribacteres* [80]–[82] were found in most of the water samples and in the green biofilms of spring 12. Sequences affiliated with *Gallionella* sp. were found uniquely in samples from spring 2, which is the only spring with favorable chemical conditions for iron oxidation (Eh >0 mV). Additionally, several species of the genus *Pelobacter*, sequences of which were found in higher frequency in the biofilms of spring 12 (0.4–1.5%), are known as S^0^ as well as iron reducers [83], [84].

Microbial iron reduction is further supported by the detection of reduced Fe in the deeper parts of the lake prior to its overturn. Nishri and Stiller [85] reported up to 4 µM Fe^2+^ in the water column prior to the 1979 Dead Sea overturn and up to 1200 µM Fe^2+^ in the pore water of the Dead Sea. During 1978–1980, following the oxygenation of the water column, reduced Fe was not detected and only particulate Fe was reported. This suggests that oxidized iron as a substrate for iron reduction is abundant in the Dead Sea sediment. Due to the abundant supply of DOC from the spring water, a community of iron reducing bacteria can be sustained.

The majority of the microbial lineages described here are known from environments of various salinities [86], usually in well established microbial mats or in overlaying waters with a constant salinity. The fluctuating nature of the Dead Sea spring system does not permit the establishment of neither a constant salinity nor a permanent gradient. Hence, a full comparison between the microbial communities in this system to those in any other system is difficult. We believe that the described flow regime prevents the growth of organisms which are adapted to a narrow range of salinities but leading to a specialized community that can cope with drastic salinity shifts.

### Environmental Fitness

Although adaptation to salinity was not investigated in this study, we expect to find two types of organisms in these environments: moderate halophiles and extreme halophiles. This is based on our underwater observations, which indicate constant and fluctuating water flow regimes. Springs 10,11, and 12 are characterized by a continuous flow with occasional changes in flow intensity. Therefore we can expect moderate halophiles to be able to grow in such a system. Indeed, the cultured green sulfur bacteria, whose 16S rRNA sequence is identical with the partial sequence of the dominant green sulfur bacteria in the biofilms of spring 12, grew best at seawater salinity (data not shown). Spring 1A, as were the large, borderless, seeping areas on the slopes below springs 10,11, and 12, were inactive for part of the time. In the center of the spring the fresher water flows directly to the surface; however, the water often seeps through the sediments at the periphery or through larger undefined areas. This suggests that the organisms in the spring sediment are exposed directly to these waters. Sudden and drastic changes in salinity, such as those induced by the onset and offset of the water flow, would call for an extreme halophile with rapidly responding osmoregulation and the ability to survive in a broad spectrum of salinities. Our data suggests that the organisms in the spring sediments are naturally found in the Dead Sea sediments; therefore an extreme halophile that can withstand freshwater would be an intriguing finding.

### Significance of the Springs for the Dead Sea

The underwater springs described here are part of one system of springs. To this date there is no information available on the number of similar spring systems on both coasts of the Dead Sea, nor about their contribution to the water budget of the Dead Sea. This is partly because deeper springs, such as those in the northern system, cannot be detected from the shore. Recent findings of Siebert and Mallast (unpublished data) indicate that thermal infrared imaging allows indirect localization of underwater springs, even deeper ones. Additional, remote sensing, studies have shown that similar systems exist on the eastern coast of the Dead Sea [87]. However, further direct underwater exploration is necessary to collect information regarding their distribution, size, and the input of water and the accompanying microbiota that these systems provide for the Dead Sea.

The economic significance of the Dead Sea lies in mineral harvesting and tourism. Both industries can suffer due to changes in the microbiology of the Dead Sea. The information about the microbial input from the springs is especially relevant in the light of the currently discussed Red Sea–Dead Sea canal. The possible outcome of diluting the Dead Sea with the Red Sea water has recently been investigated [88]. These predictions took into consideration insufficient factors: a) mixing of only two microbial communities, the residual community in the Dead Sea water column and the microbial community in the water of the Gulf of Aqaba; b) the nutrient input from the planned desalination plant. If such a water connection will be formed and the brine of the proposed desalination plant will flow into the Dead Sea, a water lens with a relatively low salinity will form on the surface of the Dead Sea. Bacteria, organic matter and other nutrients from the underwater springs are rapidly transported to the less saline water layer at the surface. This may lead to massive blooms of either spring or Dead Sea bacteria. Such blooms will alter the visual appearance of the water thus directly affecting it’s touristic value. Furthermore, the lowered salinity and increased microbial activity may promote biofilm growth and thus lead to biofouling of the equipment used by the Dead Sea Works company to pump water from the northern basin to the southern basin. We suggest that the previously conducted experiments [74] be complemented with the addition of spring water as part of the assessment of the environmental impact of the proposed Dead Sea-Red Sea water canal.

We hypothesize that the dense hydrogeochemical network of channels and the associated microbial communities in association play an important role in the overall land stability in the area. It is likely that mineral dissolution by freshwater alone is not solely responsible for the formation of the large sinkholes on the coasts of the Dead Sea [89]. Microbial activity such as sulfate reduction and oxidation leads to faster dissolution of gypsum and carbonate layers via processes know from sulfuric acid speleogenesis [90]. Sulfate released from anhydrite either by dissolution or by bacterial leaching [90], [91] is used for sulfate reduction. The produced sulfide is oxidized by bacteria into sulfuric acid which leads to the dissolution of carbonate minerals [90]. Sulfide oxidizing *Epsilonproteobacteria* as found in the water of springs 10 and 11 were suggested to be involved in sulfuric acid speleogenesis [50], [92]. Our data indicates that dense microbial communities may exist in the area between the graben shoulder and the Dead Sea. The bacterial activity coupled with the freshwater flow may lead to the enlargement of channels and the formation of underground cavities, locations where the overlaying soil may become unstable.

### Conclusions and Outlook

We have found a new microbial ecosystem in the Dead Sea, tightly associated with underwater springs that emerge at the lake floor at depths between 10–30 m. The ecosystem is diverse, and the presence of multiple major biogeochemical pathways is indicated. However, as demonstrated by our data, the springs do not serve as an input of this diversity, but more likely as a source of nourishment for the native Dead Sea community. The dominat microroganizms in the ecosystem as implicated by our microbial community and water chemistry data are phototrophs, sulfide oxidizers, sulfate reducers, nitrifiers and iron reducers. However, direct measurements of the rates of these processes are necessary to better understand the function of this microbial ecosystem and its impact on the Dead Sea ecosystem as a whole. Also detailed measurements of salinity, iron, nutrients, oxygen and stable isotopes of δ^34^S and δ^18^O in sulfate and sulfides in water as well as in and near the benthic communities are needed to elucidate the nature of the biogeochemical processes. Our data further suggest the existence of a dense microbial community within the DSG sediments and mineral bed located between the former and current Dead Sea water level line. However, the activity of this community and their role in the dissolution of minerals in these sediments remain to be determined. The underwater system of springs described here is the first out of several in the Dead Sea. Together, they are an unknown factor in its water budget as well as an unknown source of diversity and metabolic potential.

## Supporting Information

Figure S1 A) Lamination on the walls of the shafts created by the springs in the northern system. B) An example of a single water source out of several at the bottom of a shaft in the northern system. C) An example of an in-shaft cavity from which water springs out. D) Cobble covered spring in the southern system. Biofillms are visible on the cobble. Scale bar 0.2 m.
(TIF)

Figure S2 **Different types of biofilms found near the underwater springs.** (A) Small patches of thin white biofilms covered sediments adjacent to the water source in springs 1–5. (B) Thick white biofilms covered sediments around spring 12, whereas top and bottom surfaces of rocks found within this spring were covered with green and white biofilms, respectively. (C) Large white biofilms covered slopes below springs 10 and 11 at depths ca. 20 m, although no water seepage was detected. Scale bar: 0.2 m
(TIF)

Figure S3 **Chlorophyll a autofluorescence confocal laser scanning microscopy of samples from the green biofilms of spring 12 showing small unicellular cyanobacteria (A) and diatoms (B).** The images were acquired by Mr. Assaf Lowenthal.
(TIF)

Figure S4 **Rarefaction curves for the different sediment, biofilm and water samples, as derived from the pyrosequencing data by the NGS pipeline.** Samples names are given at the end of the curve: W, S, WB, GB stand for water, sediment, white biofilm and green biofilm respectively, followed by the spring number.
(TIF)

Figure S5 **Comparison of underwater spring waters, sediments and biofilms based on the taxonomy assigned to the pyrosequecnig results at the 6^th^ taxonomic depth.** Panel A considers only sequence identity, whereas panel B takes additionally into account sequence frequency. The diagrams show that the reference microbial community shared 45% of the taxa with the spring sediments (expect of spring 11) as opposed to 10% with the spring waters. The similarity between the reference sediments and springs sediments increased 50%–70% when sequence frequency was additionally taken into account, with the exception of spring 11. The reference Dead Sea sediments were collected in the northern spring system, whereas spring 11 belongs to the southern system
(TIF)

Figure S6 **Percent abundance of total Bacteria,** ***Epsilonproteobacteria*** **,** ***Cytofaga*** **/** ***Flavobacteria*** **and** ***Deltaproteobacteria*** **out of total DAPI stained cells as obtained by Fluorescent** ***In-Situ*** **Hybridization on a white biofilm from spring 12.** FISH was conducted using the EUBI, II, II, Eps404, CF119a and Delta* 495a, b, c probes. *Competitor probes were used. Total cell number as calculated from the cell counts is 2.8×10^10^ cells g^−1^.
(TIF)

Figure S7 **Calculated REY concentrations based on the mixing coefficients in** **Table 2** **using the JGA as a source and the Dead Sea (A) and Qedem brine (B) as admixing waters.** Extracted porewater was not sufficient to measure REY concentration, hence, Dead Sea water was used instead for the purpose of this calculation alone. The ratio between the measured REY concentration and those calculated with Dead Sea water (C) and Qedem brine (D) show a clear separation between the “Limestone” group (full lines) and the “Dead Sea” group (dashed line). The flat lines of the “Limestone” group suggest mainly mixing is involved, whereas the decreasing lines of the “Dead Sea” group point to the involvement of other processes in the determination of the REY pattern. The three water source, JGA, Dead Sea and Qedem brine are shown in full line with symbols.
(TIF)

Table S1 **REY concentration in sampled waters and whole rock analyses. Tm is not listed, since it was added as spike.**
(DOCX)

Table S2 **Sequence statistics per sample as obtained from the NGS analysis:** #Seqs :The number of reads in this sample; Min: The shortest read in this sample (number of nucleotides); Max: The longest read in this sample (number of nucleotides); Average: The average length of a read in this sample (number of nucleotides); Rejected: Number of reads rejected by the aligner (possible contamination); Homopolymer: Number of reads rejected by the quality control because of a problematic amount of homopolymeric stretches in the read; Clustered: The number of reads assigned to a cluster within the same sample (98% identity); Replicates: The number of reads identical to another read within the the sample sample (100% identity); False Hit: The number of reads outside the target group of the used primers; OTUs (total): The total number of unique reads, at 98% sequence similarity, in each sample; OTUs (class.): The number of unique reads with an assigned taxonomic classification; #Seqs (class.): The total number of reads with an assigned taxonomic classification; OTUs (unclass.): The number of unique reads without an assigned taxonomic classification; #Seqs (unclass.): The total number of reads without an assigned taxonomic classification. Good’s coverage [94] was calculated as 1– (n_i_/N) where n_i_ is the number of OTUs containing only one sequence and N is the total number of sequences. The false positive Bacterial sequences obtained while using Archaea specific primers, were not used for the calculation.
(DOCX)

Table S3 **A list of Bacterial taxonomic paths and their relative abundance in the samples.** Where possible, the taxonomic path is resolved down to the genus level (depth 6). The table is available as a separate file.
(XLS)

Table S4 **A list of Archaeal taxonomic paths and their relative abundance in the samples.** Where possible, the taxonomic path is resolved down to the genus level (depth 6). The list contains also the Bacterial (Bacteria*) sequences obtained using the Archaeal specific primers. The table is available as a separate file
(XLS)

Table S5 **Analysis of similarity (ANOSIM) of the 454 (A) and ARISA (B) data using the DICE algorithm as implemented in the PAST software.** R and p values are shown in the lower and upper half of the square matrix respectively. An R value of 1 between two groups represents total dissimilarity, whereas values closer to 0 suggest a high similarity. The significance of the similarity analysis (p) was done by permutation of group memberships using 10,000 replicates. P values of significance are marked in bold and italics.
(DOCX)

Table S6 Trace element concentrations in sampled waters. Si* is the sum of Si(II) and Si(III). ** Mn is the total sum of all Mn-species. Saturation indices were calculated by Geochemist’s Workbench (using LLNL thermo database and Harvie-Møller-Weare activity model, as implemented in the USGS program PHREEQPITZ.
(DOCX)
