# Supplementary figures and images for: Microbial and Chemical Characterization of Underwater Fresh Water Springs in the Dead Sea
Source: PLoS One. 2012 Jun 5;7(6):e38319. doi: 10.1371/journal.pone.0038319 (PMC3367964; doi:10.1371/journal.pone.0038319)

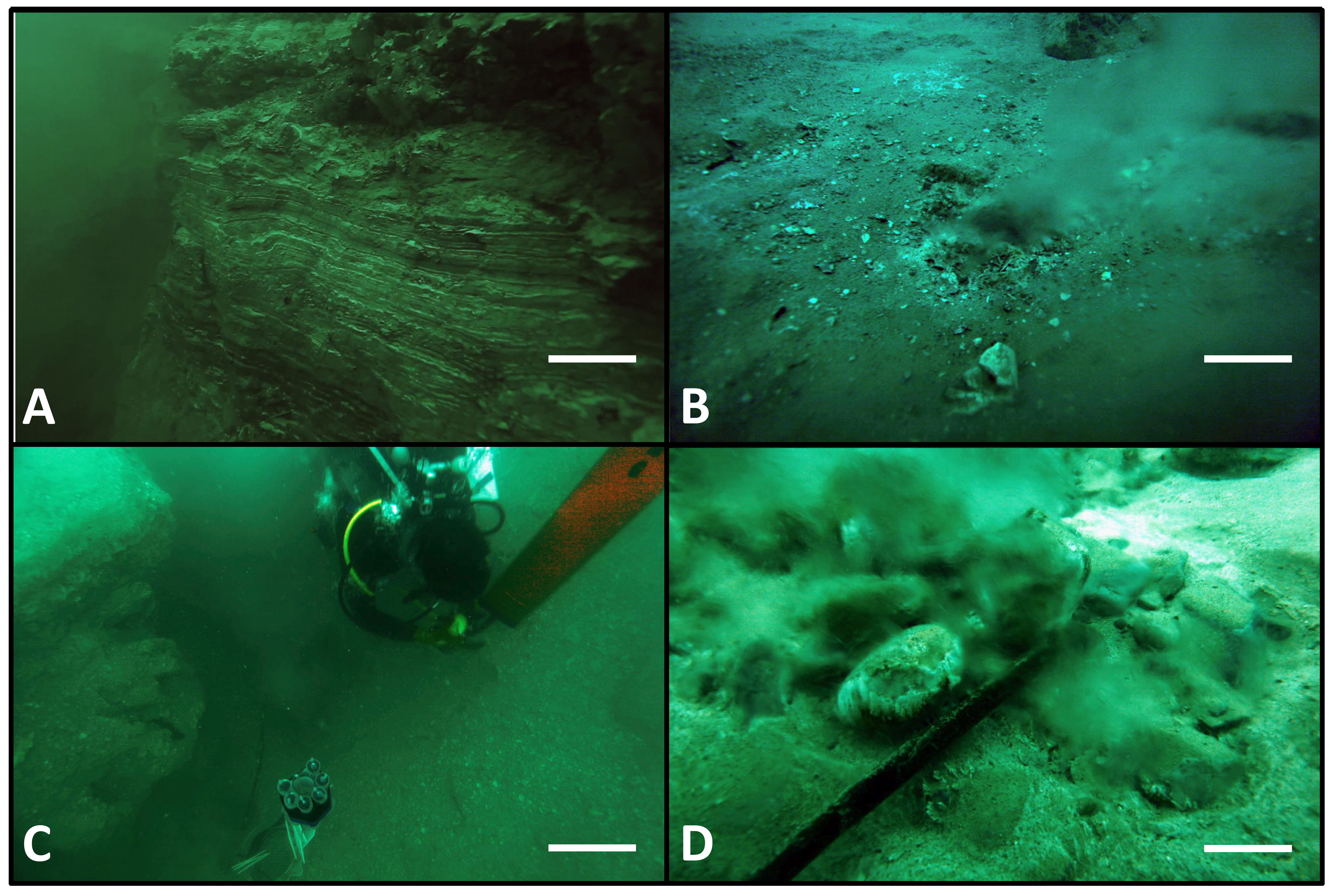

Supplement: Figure S1 — A) Lamination on the walls of the shafts created by the springs in the northern system. B) An example of a single water source out of several at the bottom of a shaft in the northern system. C) An example of an in-shaft cavity from which water springs out. D) Cobble covered spring in the southern system. Biofillms are visible on the cobble. Scale bar 0.2 m. (TIF) [file pone.0038319.s001.tif]

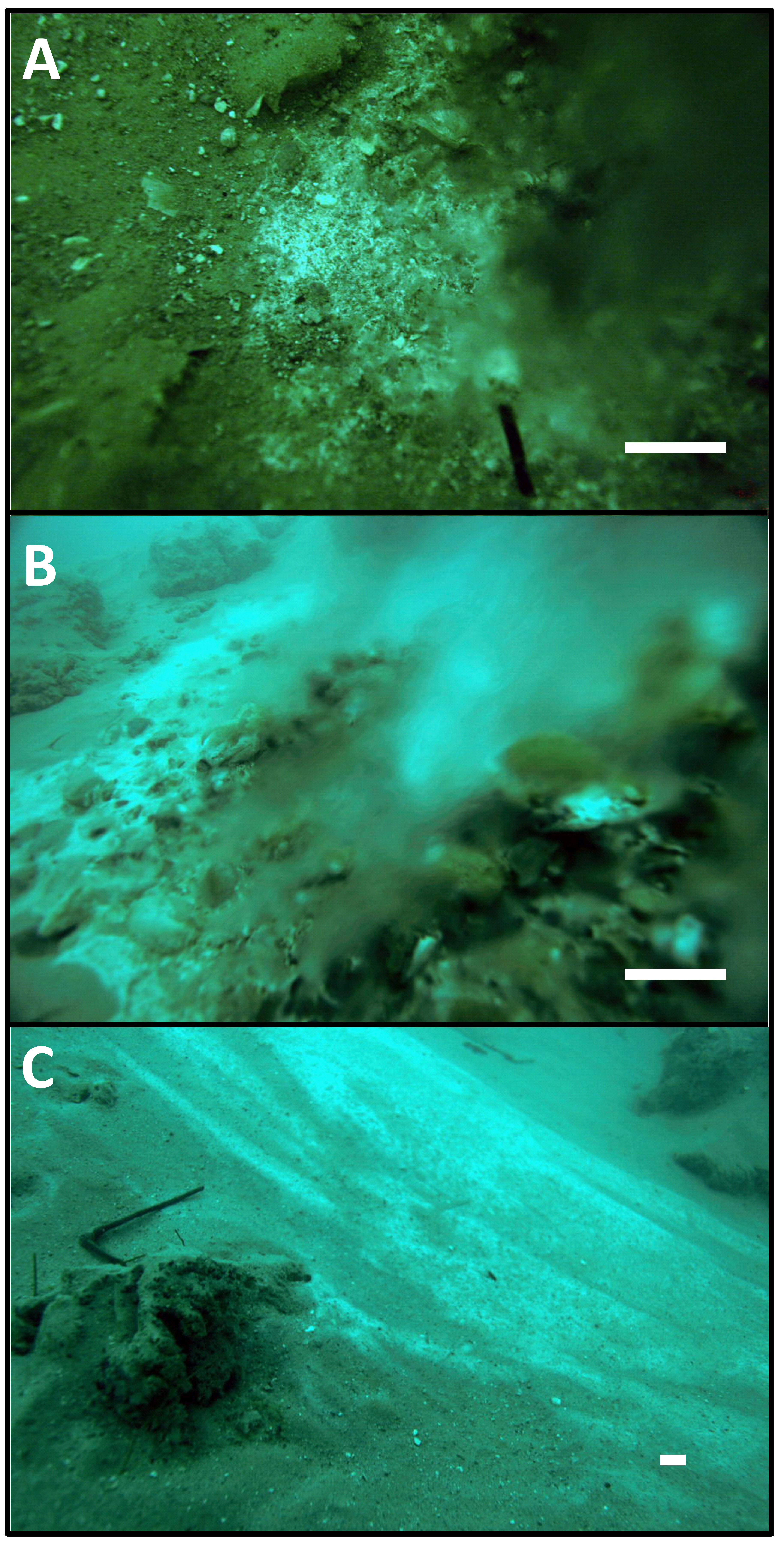

Supplement: Figure S2 — Different types of biofilms found near the underwater springs. (A) Small patches of thin white biofilms covered sediments adjacent to the water source in springs 1–5. (B) Thick white biofilms covered sediments around spring 12, whereas top and bottom surfaces of rocks found within this spring were covered with green and white biofilms, respectively. (C) Large white biofilms covered slopes below springs 10 and 11 at depths ca. 20 m, although no water seepage was detected. Scale bar: 0.2 m (TIF) [file pone.0038319.s002.tif]

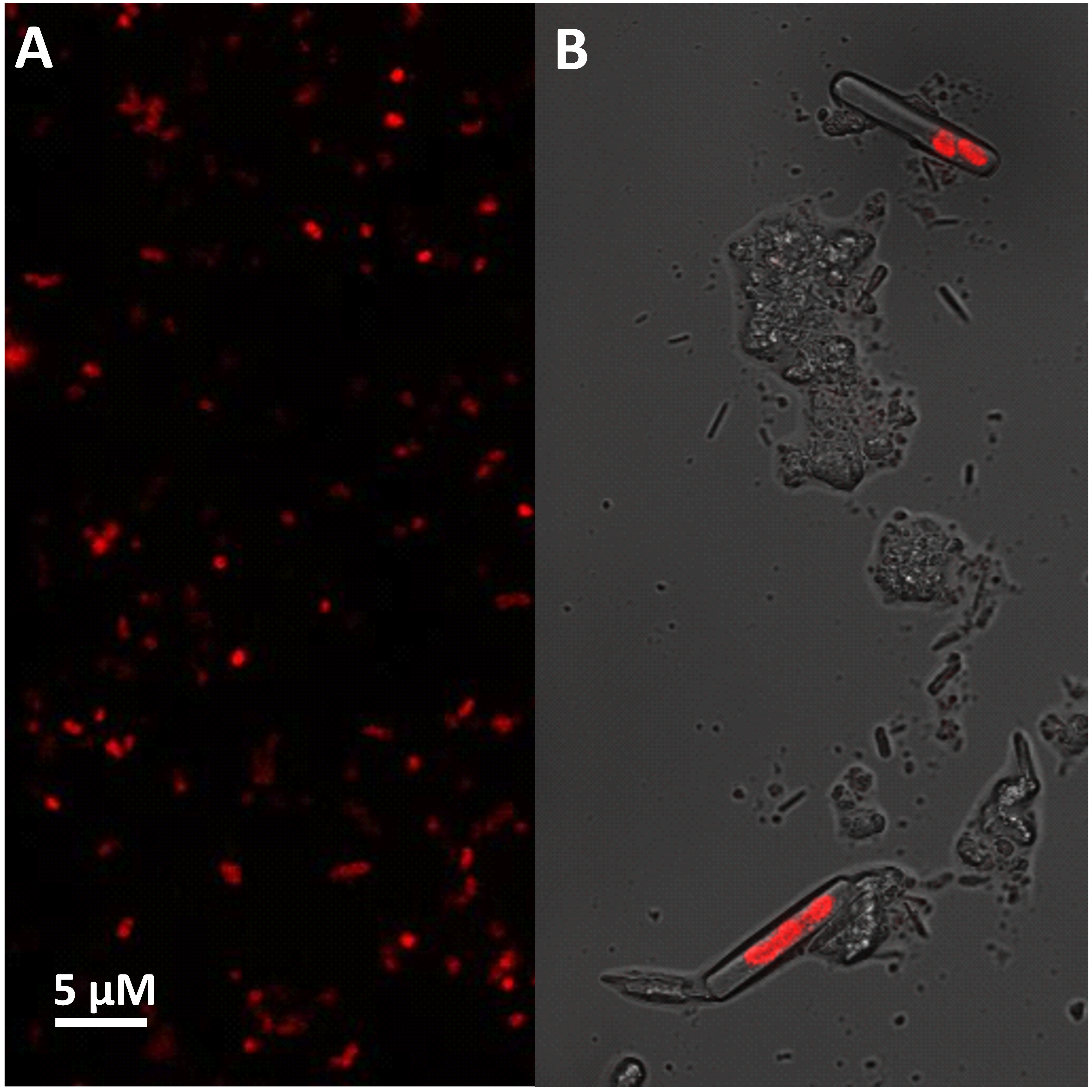

Supplement: Figure S3 — Chlorophyll a autofluorescence confocal laser scanning microscopy of samples from the green biofilms of spring 12 showing small unicellular cyanobacteria (A) and diatoms (B). The images were acquired by Mr. Assaf Lowenthal. (TIF) [file pone.0038319.s003.tif]

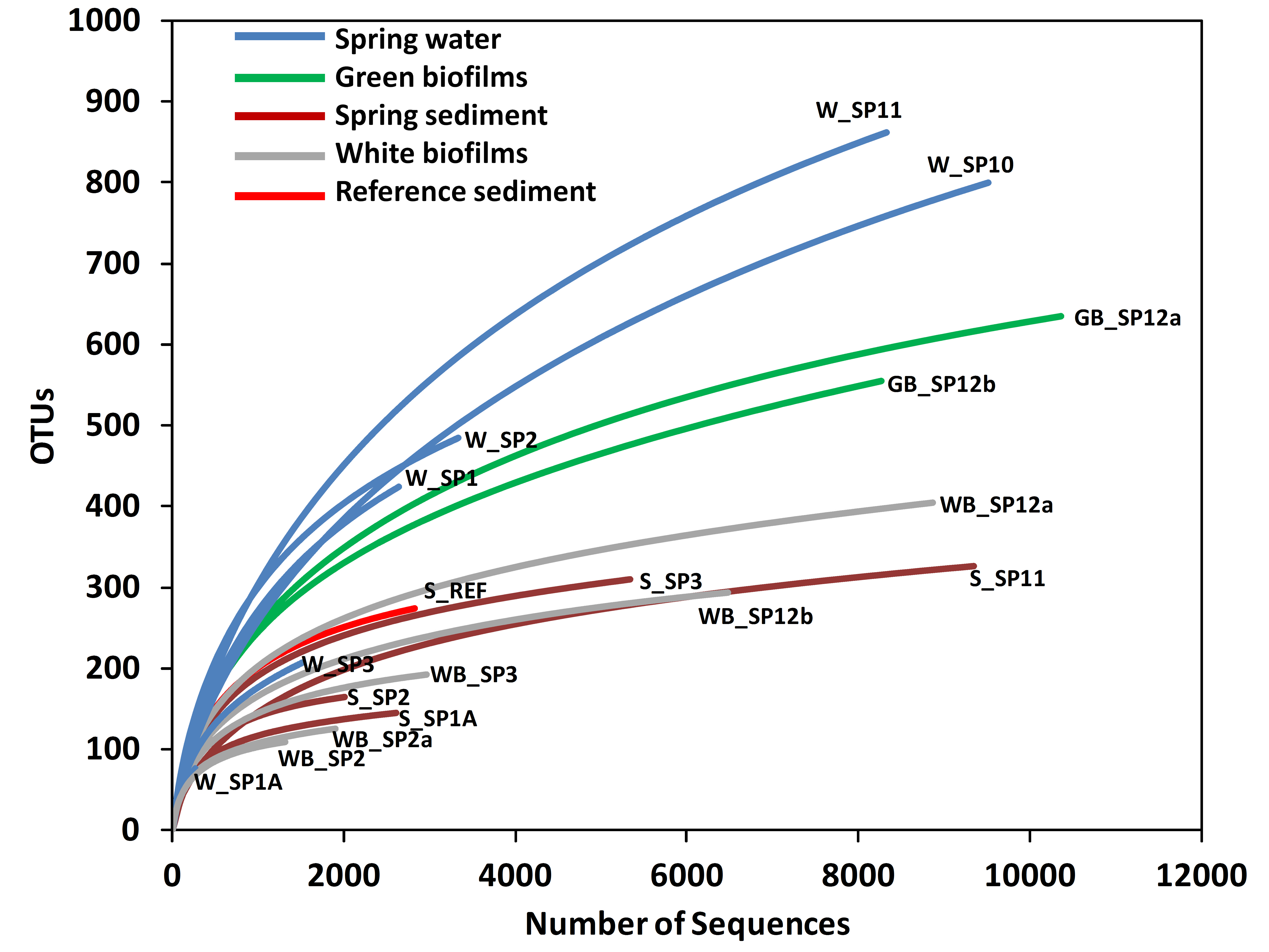

Supplement: Figure S4 — Rarefaction curves for the different sediment, biofilm and water samples, as derived from the pyrosequencing data by the NGS pipeline. Samples names are given at the end of the curve: W, S, WB, GB stand for water, sediment, white biofilm and green biofilm respectively, followed by the spring number. (TIF) [file pone.0038319.s004.tif]

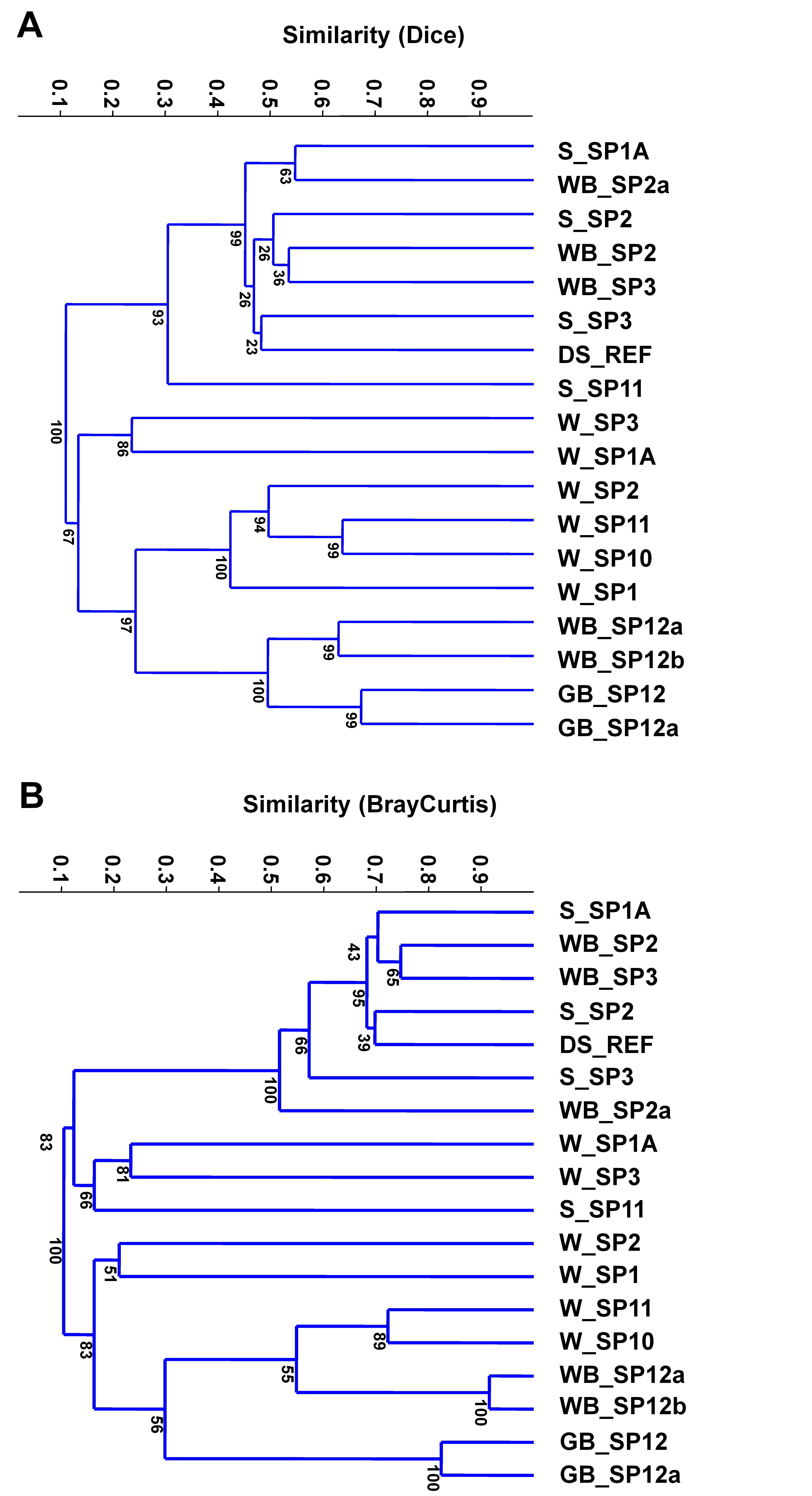

Supplement: Figure S5 — Comparison of underwater spring waters, sediments and biofilms based on the taxonomy assigned to the pyrosequecnig results at the 6th taxonomic depth. Panel A considers only sequence identity, whereas panel B takes additionally into account sequence frequency. The diagrams show that the reference microbial community shared 45% of the taxa with the spring sediments (expect of spring 11) as opposed to 10% with the spring waters. The similarity between the reference sediments and springs sediments increased 50%–70% when sequence frequency was additionally taken into account, with the exception of spring 11. The reference Dead Sea sediments were collected in the northern spring system, whereas spring 11 belongs to the southern system (TIF) [file pone.0038319.s005.tif]

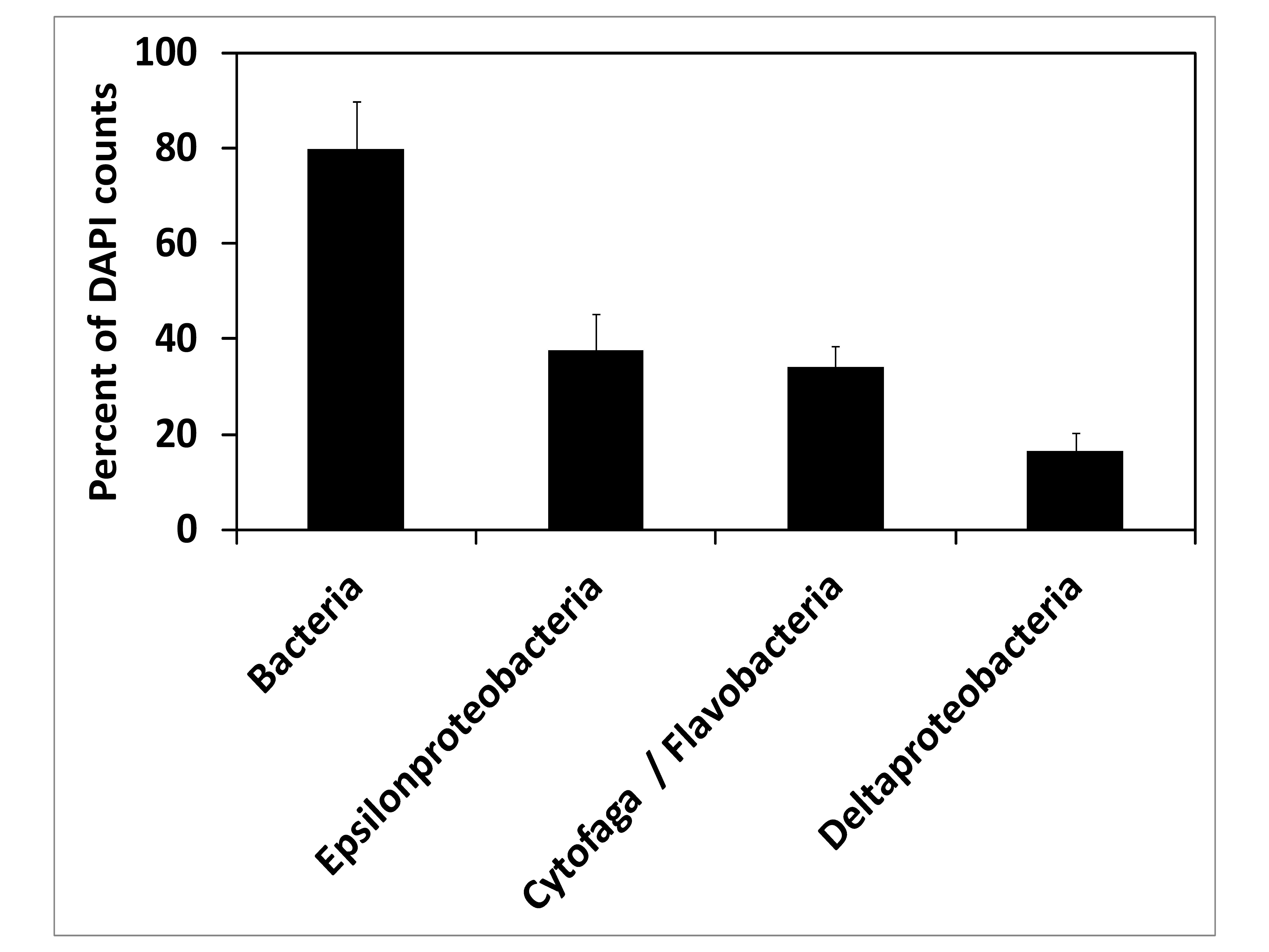

Supplement: Figure S6 — Percent abundance of total Bacteria, Epsilonproteobacteria , Cytofaga / Flavobacteria and Deltaproteobacteria out of total DAPI stained cells as obtained by Fluorescent In-Situ Hybridization on a white biofilm from spring 12. FISH was conducted using the EUBI, II, II, Eps404, CF119a and Delta* 495a, b, c probes. *Competitor probes were used. Total cell number as calculated from the cell counts is 2.8×10^10^ cells g−1. (TIF) [file pone.0038319.s006.tif]

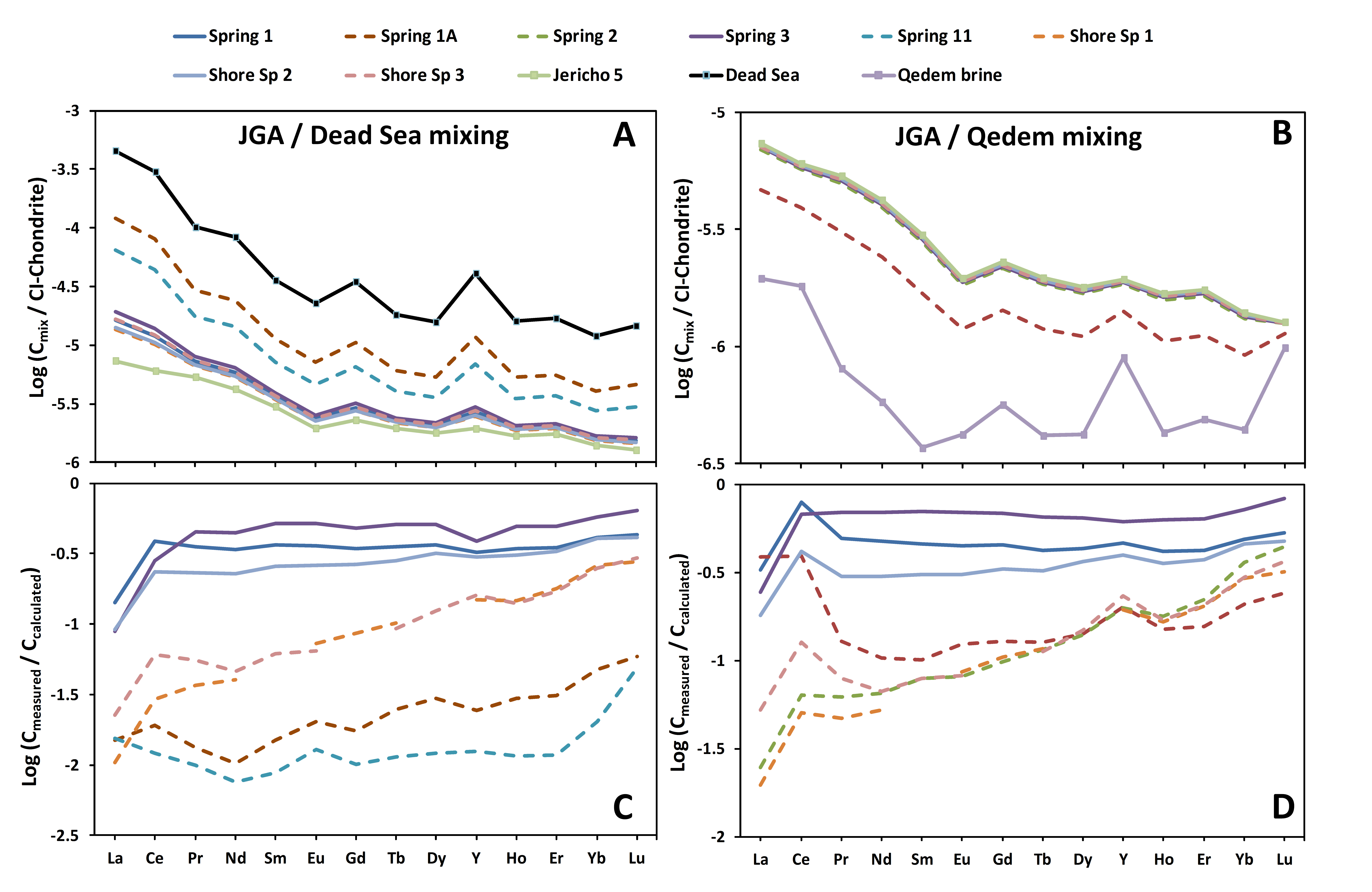

Supplement: Figure S7 — Calculated REY concentrations based on the mixing coefficients in Table 2 using the JGA as a source and the Dead Sea (A) and Qedem brine (B) as admixing waters. Extracted porewater was not sufficient to measure REY concentration, hence, Dead Sea water was used instead for the purpose of this calculation alone. The ratio between the measured REY concentration and those calculated with Dead Sea water (C) and Qedem brine (D) show a clear separation between the “Limestone” group (full lines) and the “Dead Sea” group (dashed line). The flat lines of the “Limestone” group suggest mainly mixing is involved, whereas the decreasing lines of the “Dead Sea” group point to the involvement of other processes in the determination of the REY pattern. The three water source, JGA, Dead Sea and Qedem brine are shown in full line with symbols. (TIF) [file pone.0038319.s007.tif]
