# Supplementary material for: Microbial and Chemical Characterization of Underwater Fresh Water Springs in the Dead Sea
Source: PLoS One. 2012 Jun 5;7(6):e38319. doi: 10.1371/journal.pone.0038319 (PMC3367964; doi:10.1371/journal.pone.0038319)
Supplement: Table S1 — REY concentration in sampled waters and whole rock analyses. Tm is not listed, since it was added as spike. (DOCX) [file pone.0038319.s008.docx]

# *Table S1:*

| **pmol/L** | **La** | **Ce** | **Pr** | **Nd** | **Sm** | **Eu** | **Gd** | **Tb** | **Dy** | **Y** | **Ho** | **Er** | **Yb** | **Lu** |
| --- | --- | --- | --- | --- | --- | --- | --- | --- | --- | --- | --- | --- | --- | --- |
| **Spring 1** | 3.06 | 6.59 | 0.25 | 0.78 | 0.17 | 0.05 | 0.23 | 0.03 | 0.23 | 5.00 | 0.05 | 0.17 | 0.18 | 0.04 |
| **Spring 1A** | 3.92 | 20.0 | 1.62 | 6.09 | 1.30 | 0.31 | 1.27 | 0.18 | 1.13 | 15.4 | 0.23 | 0.68 | 0.62 | 0.09 |
| **Spring 2** | 0.29 | 1.56 | 0.19 | 0.80 | 0.22 | 0.05 | 0.27 | 0.05 | 0.35 | 6.43 | 0.10 | 0.35 | 0.45 | 0.08 |
| **Spring 3** | 2.91 | 16.9 | 2.25 | 8.79 | 1.95 | 0.48 | 1.89 | 0.28 | 1.66 | 20.4 | 0.35 | 1.02 | 0.91 | 0.14 |
| **Spring 11** | 1.70 | 2.29 | 0.11 | 0.34 | 0.06 | 0.02 | 0.08 | 0.01 | 0.06 | 1.52 | 0.01 | 0.04 | 0.05 | 0.02 |
| **Shore Spr. 1** | 0.66 | 2.23 | 0.20 | 0.73 | 0.19 | 0.05 | BDL* | 0.05 | 0.40 | 7.10 | 0.10 | 0.32 | 0.38 | 0.06 |
| **Shore Spr. 2** | 2.18 | 10.6 | 0.99 | 3.86 | 0.87 | 0.22 | 0.93 | 0.14 | 0.95 | 13.2 | 0.20 | 0.61 | 0.59 | 0.08 |
| **Shore Spr. 3** | 3.65 | 16.1 | 2.15 | 8.59 | 2.03 | 0.50 | 2.12 | 0.34 | 1.90 | 22.7 | 0.39 | 1.11 | 1.00 | 0.15 |
| **Qedem Brine** | 3.30 | 7.78 | 0.51 | 1.82 | 0.36 | 0.16 | 0.71 | 0.09 | 0.63 | 15.7 | 0.15 | 0.46 | 0.42 | 0.14 |
| **Auja 2** | 3.08 | 6.11 | 0.71 | 2.45 | 0.54 | 0.15 | 0.54 | 0.08 | 0.55 | 7.20 | 0.11 | 0.33 | 0.37 | 0.05 |
| **Auja 4** | 2.18 | 4.03 | 0.44 | 1.24 | 0.33 | 0.08 | 0.35 | 0.05 | 0.38 | 5.30 | 0.08 | 0.25 | 0.28 | 0.03 |
| **Jericho 5** | 12.4 | 25.9 | 3.37 | 13.2 | 2.92 | 0.72 | 2.87 | 0.44 | 2.67 | 34.0 | 0.57 | 1.66 | 1.31 | 0.17 |
| **Mitzpe Jericho 2** | 3.26 | 4.29 | 0.63 | 2.19 | 0.60 | 0.15 | 0.72 | 0.10 | 0.83 | 16.8 | 0.22 | 0.75 | 0.81 | 0.14 |
| **HammatGader: EinMakla** | 10.3 | 14.7 | 1.81 | 7.43 | 1.28 | 0.38 | 1.83 | 0.31 | 1.99 | 47.0 | 0.48 | 1.84 | 1.92 | 0.33 |
| **EinQilt 1** | 43.5 | 66.5 | 11.9 | 58.3 | 13.3 | 3.94 | 20.2 | 3.32 | 28.0 | 530 | 8.36 | 29.1 | 24.7 | 3.76 |
| **EinQilt 2** | 43.7 | 28.7 | 12.1 | 59.1 | 13.2 | 3.92 | 20.6 | 3.35 | 28.4 | 552 | 8.46 | 29.6 | 25.2 | 3.82 |
| **Lake Kinneret, hypolimnion** | 17.1 | 42.9 | 3.68 | 15.4 | 2.93 | 0.80 | 3.38 | 0.49 | 3.18 | 61.8 | 0.74 | 2.35 | 2.15 | 0.36 |
| **µg/g** |  |  |  |  |  |  |  |  |  |  |  |  |  |  |
| **Limestone (JGA)** | 17.1 | 10.5 | 2.59 | 11.0 | 2.39 | 0.61 | 3.43 | 0.51 | 3.54 | 48.1 | 0.90 | 3.04 | 3.12 | 0.53 |
| **Marlstone (JGA)** | 1.39 | 0.59 | 0.24 | 0.96 | 0.18 | 0.05 | 0.21 | 0.03 | 0.17 | 2.11 | 0.04 | 0.12 | 0.09 | 0.01 |
