# Supplementary material for: Microbial and Chemical Characterization of Underwater Fresh Water Springs in the Dead Sea
Source: PLoS One. 2012 Jun 5;7(6):e38319. doi: 10.1371/journal.pone.0038319 (PMC3367964; doi:10.1371/journal.pone.0038319)
Supplement: Table S2 — Sequence statistics per sample as obtained from the NGS analysis: #Seqs :The number of reads in this sample; Min: The shortest read in this sample (number of nucleotides); Max: The longest read in this sample (number of nucleotides); Average: The average length of a read in this sample (number of nucleotides); Rejected: Number of reads rejected by the aligner (possible contamination); Homopolymer: Number of reads rejected by the quality control because of a problematic amount of homopolymeric stretches in the read; Clustered: The number of reads assigned to a cluster within the same sample (98% identity); Replicates: The number of reads identical to another read within the the sample sample (100% identity); False Hit: The number of reads outside the target group of the used primers; OTUs (total): The total number of unique reads, at 98% sequence similarity, in each sample; OTUs (class.): The number of unique reads with an assigned taxonomic classification; #Seqs (class.): The total number of reads with an assigned taxonomic classification; OTUs (unclass.): The number of unique reads without an assigned taxonomic classification; #Seqs (unclass.): The total number of reads without an assigned taxonomic classification. Good’s coverage [94] was calculated as 1– (ni/N) where ni is the number of OTUs containing only one sequence and N is the total number of sequences. The false positive Bacterial sequences obtained while using Archaea specific primers, were not used for the calculation. (DOCX) [file pone.0038319.s009.docx]

## Table S2:

|  | | **General** | | | | | | | | | | **Classified** | | **Unclassified** | | **Good’s coverage** |
| --- | --- | --- | --- | --- | --- | --- | --- | --- | --- | --- | --- | --- | --- | --- | --- | --- |
|  | **Sample Name** | **#Seqs** | **Min** | **Average** | **Max** | **Rejected** | **Homopolymer** | **Clustered** | **Replicate** | **False Hits** | **OTUs** | **OTUs** | **#Seqs** | **OTUs** | **#Seqs** |  |
| BACTERIA | W SP1 | 2695 | 200 | 452.5988 | 561 | 0 | 0 | 1822 | 427 | 0 | 446 | 446 | 2695 | 0 | 0 | 0.977 |
|  | W SP1A | 262 | 200 | 444.5701 | 536 | 0 | 0 | 147 | 37 | 0 | 78 | 78 | 262 | 0 | 0 | 0.989 |
|  | W SP2 | 3632 | 201 | 450.495 | 557 | 0 | 3 | 2651 | 372 | 0 | 606 | 603 | 3629 | 3 | 3 | 0.933 |
|  | W SP3 | 1528 | 200 | 348.9996 | 539 | 0 | 0 | 1145 | 171 | 0 | 212 | 212 | 1528 | 0 | 0 | 0.994 |
|  | W SP10 | 10073 | 200 | 469.3248 | 561 | 0 | 6 | 7252 | 1728 | 0 | 1087 | 1078 | 10033 | 9 | 40 | 0.948 |
|  | WSP11 | 8811 | 200 | 469.1671 | 559 | 2 | 8 | 6663 | 1072 | 0 | 1066 | 1056 | 8771 | 10 | 40 | 0.949 |
|  | S SP1A | 2653 | 150 | 354.3404 | 499 | 0 | 8 | 1678 | 814 | 0 | 153 | 150 | 2649 | 3 | 4 | 0.986 |
|  | S SP2 | 2078 | 150 | 417.9216 | 547 | 5 | 26 | 1310 | 562 | 0 | 175 | 170 | 2048 | 5 | 30 | 0.981 |
|  | S SP3 | 5449 | 150 | 421.0319 | 651 | 0 | 51 | 3251 | 1804 | 0 | 343 | 327 | 5423 | 16 | 26 | 0.981 |
|  | S SP11 | 9426 | 150 | 381.9789 | 545 | 0 | 8 | 4137 | 4922 | 0 | 359 | 300 | 9187 | 59 | 239 | 0.990 |
|  | WB SP2 | 1363 | 150 | 418.2406 | 541 | 0 | 16 | 856 | 371 | 0 | 120 | 119 | 1362 | 1 | 1 | 0.979 |
|  | WB SP3 | 3060 | 150 | 416.7288 | 555 | 0 | 36 | 1850 | 959 | 0 | 215 | 204 | 3025 | 11 | 35 | 0.981 |
|  | WB SP2a | 1940 | 150 | 351.1103 | 491 | 0 | 9 | 1203 | 589 | 0 | 139 | 136 | 1937 | 3 | 3 | 0.981 |
|  | WB SP12a | 8973 | 200 | 469.4033 | 545 | 0 | 0 | 7096 | 1423 | 0 | 454 | 454 | 8973 | 0 | 0 | 0.984 |
|  | WB SP12b | 6541 | 200 | 468.8662 | 545 | 0 | 0 | 5097 | 1115 | 0 | 329 | 329 | 6541 | 0 | 0 | 0.985 |
|  | GB SP12a | 10509 | 200 | 455.5848 | 539 | 0 | 0 | 7538 | 2273 | 0 | 698 | 697 | 10506 | 1 | 3 | 0.979 |
|  | GB SP12b | 8408 | 200 | 451.8204 | 543 | 2 | 1 | 6228 | 1554 | 0 | 623 | 621 | 8401 | 2 | 7 | 0.972 |
|  | S DS | 2919 | 151 | 418.5074 | 545 | 3 | 32 | 1801 | 788 | 0 | 295 | 288 | 2908 | 7 | 11 | 0.971 |
| ARCHAEA | W SP1 | 6313 | 200 | 416.7375 | 589 | 1 | 15 | 4703 | 521 | 1245 | 1073 | 1064 | 6299 | 9 | 14 | 0.930 |
|  | W SP1A | 1864 | 201 | 422.2135 | 487 | 0 | 1 | 1533 | 195 | 628 | 135 | 135 | 1864 | 0 | 0 | 0.985 |
|  | W SP2 | 6200 | 200 | 413.0065 | 514 | 1 | 54 | 5025 | 497 | 974 | 623 | 620 | 6195 | 3 | 5 | 0.968 |
|  | W SP3 | 6754 | 200 | 415.712 | 558 | 1 | 56 | 5568 | 637 | 1452 | 492 | 487 | 6747 | 5 | 7 | 0.994 |
|  | S SP1A | 1336 | 154 | 432.5157 | 498 | 0 | 0 | 908 | 395 | 416 | 33 | 33 | 1336 | 0 | 0 | 0.996 |
|  | S SP2 | 3148 | 150 | 417.8796 | 488 | 0 | 0 | 1672 | 1404 | 2785 | 72 | 71 | 3147 | 1 | 1 | 0.990 |
|  | S SP3 | 1665 | 152 | 419.8246 | 498 | 0 | 0 | 989 | 631 | 0 | 45 | 45 | 1665 | 0 | 0 | 0.994 |
|  | WB SP2a | 1898 | 150 | 397.5479 | 452 | 0 | 0 | 1153 | 710 | 1603 | 35 | 35 | 1898 | 0 | 0 | 0.996 |
|  | GB SP12a | 929 | 206 | 407.578 | 475 | 0 | 0 | 685 | 201 | 21 | 43 | 43 | 929 | 0 | 0 | 0.988 |
|  | GB SP12b | 10423 | 200 | 414.6396 | 529 | 0 | 0 | 6138 | 3945 | 2302 | 340 | 340 | 10423 | 0 | 0 | 0.990 |
|  | S DS ARCH | 581 | 153 | 427.3563 | 492 | 0 | 0 | 430 | 129 | 578 | 22 | 22 | 581 | 0 | 0 | 0.989 |
