# Supplementary material for: Microbial and Chemical Characterization of Underwater Fresh Water Springs in the Dead Sea
Source: PLoS One. 2012 Jun 5;7(6):e38319. doi: 10.1371/journal.pone.0038319 (PMC3367964; doi:10.1371/journal.pone.0038319)
Supplement: Table S5 — Analysis of similarity (ANOSIM) of the 454 (A) and ARISA (B) data using the DICE algorithm as implemented in the PAST software. R and p values are shown in the lower and upper half of the square matrix respectively. An R value of 1 between two groups represents total dissimilarity, whereas values closer to 0 suggest a high similarity. The significance of the similarity analysis (p) was done by permutation of group memberships using 10,000 replicates. P values of significance are marked in bold and italics. (DOCX) [file pone.0038319.s012.docx]

## Table S5

**A**

| **R P** | **Water North** | **Water South** | **Sediment** | **WB North** | **WB South** | **GB South** |
| --- | --- | --- | --- | --- | --- | --- |
| **Water North** |  | 0.1327 | ***0.0151*** | ***0.0295*** | 0.0684 | 0.0657 |
| **Water South** | **0.2143** |  | ***0.0503*** | 0.0949 | 0.3327 | 0.3302 |
| **Sediment** | **0.7625** | **1** |  | 0.6028 | ***0.0466*** | ***0.0454*** |
| **WB North** | **1** | **1** | **0.09744** |  | 0.103 | 0.104 |
| **WB South** | **0.5893** | **1** | **1** | **1** |  | 0.338 |
| **GB South** | **0.4286** | **1** | **1** | **1** | **1** |  |

**B**

| **R P** | **Sediments** | **Water 1A, 3** | **Water 1, 10, 11** |
| --- | --- | --- | --- |
| **Sediments** |  | ***0.0032*** | ***0.0006*** |
| **Water 1A, 3** | **0.9335** |  | 0.6029 |
| **Water 1, 10, 11** | **0.993** | **0** |  |
