# Supplementary material for: Microbial and Chemical Characterization of Underwater Fresh Water Springs in the Dead Sea
Source: PLoS One. 2012 Jun 5;7(6):e38319. doi: 10.1371/journal.pone.0038319 (PMC3367964; doi:10.1371/journal.pone.0038319)
Supplement: Table S6 — Trace element concentrations in sampled waters. Si* is the sum of Si(II) and Si(III). ** Mn is the total sum of all Mn-species. Saturation indices were calculated by Geochemist’s Workbench (using LLNL thermo database and Harvie-Møller-Weare activity model, as implemented in the USGS program PHREEQPITZ. (DOCX) [file pone.0038319.s013.docx]

## Table S6

|  | **Cs^+^** | **Rb^+^** | **Ba^2+^** | **B^3+^** | **Sr^2+^** | **Li^+^** | **Si*** | **Mn**** | **Saturation Indices (SI)** | | | | | | | | |
| --- | --- | --- | --- | --- | --- | --- | --- | --- | --- | --- | --- | --- | --- | --- | --- | --- | --- |
|  | **nM** | **µM** | **µM** | **mM** | **mM** | **mM** | **mM** | **µM** | **Anhy-**  **drite** | **Aragonite** | **Bar-**  **ite** | **Cal-**  **cite** | **Celes-**  **tine** | **Dolo-**  **mite** | **Gyp-**  **sum** | **Ha-lite** | **Stronti-anite** |
| **Dead Sea** | 87.4 | 26.6 | 16.2 | 3.11 | 3.89 | 2.04 | 0.08 | 50.0 | 0.657 | 0.91 | - | 1.09 | - | 3.41 | 0.516 | 0.758 | - |
| **Spring 1** | 1.30 | 0.84 | 1.82 | 0.28 | 0.18 | 0.06 | 0.66 | 0 | -1.28 | 0.769 | 0.524 | 0.934 | -0.939 | 3.34 | -1.13 | -4.84 | 1.90 |
| **Spring 1A** | 18.8 | 6.44 | 3.57 | 1.94 | 1.37 | 0.70 | 0.61 | 30.0 | -0.172 | 0.202 | - | 0.386 | - | 1.71 | 0.0365 | 2.63 | - |
| **Spring 2** | 2.62 | 1.61 | 3.06 | 0.60 | 0.35 | 0.14 | 0.72 | 0 | -1.18 | 0.840 | 0.652 | 1.00 | -0.564 | 3.55 | 1.04 | -4.31 | 2.24 |
| **Spring 3** | 1.94 | 1.25 | 3.50 | 0.40 | 0.55 | 0.08 | 0.69 | 0 | -1.47 | 1.00 | 0.439 | 1.17 | -0.923 | 3.93 | 1.40 | -4.65 | 2.31 |
| **Spring 4** | - | - | - | - | - | - | - | - | - | - | - | - | - | - | - | - | - |
| **Spring 10** | - | - | - | - | - | - | - | - | - | - | - | - | - | - | - | - |  |
| **Spring 11^#^** | 8.28 | 3.51 | 2.84 | 1.06 | 0.58 | 0.43 | 0.49 | 10 | -1.4 | - | 0.0333 | - | -1.17 | - | -1.29 | -3.41 | -1.40 |
| **Sh Spring 1** | 2.11 | 0.60 | 2.62 | 0.16 | 0.10 | 0.03 | 0.31 | 0 | -1.71 | 0.550 | 0.223 | 0.715 | -1.52 | 2.90 | -1.56 | -5.14 | 1.53 |
| **Sh Spring 2** | 1.17 | 0.62 | 1.52 | 0.24 | 0.12 | 0.04 | 0.47 | 0 | -1.89 | 0.756 | 0.189 | 0.920 | -1.69 | 3.32 | -1.73 | -5.04 | 1.75 |
| **Sh Spring 3** | 1.25 | 0.54 | 1.38 | 0.20 | 0.15 | BDL | 0.37 | 0 | -1.76 | 0.501 | 0.301 | 0.665 | -1.68 | 2.82 | -1.61 | -4.90 | 1.38 |
| **Pore water** | 130.2 | 36.3 | 7.60 | 3.57 | 7.61 | 2.38 | 0.22 | 460 | 0.225 | 0.0262 | - | 0.156 | - | 1.64 | 0.0099 | -0.537 | - |
| **Qedem brine** | 138.5 | 15.9 | 6.70 | 2.56 | 3.94 | 1.30 | 0.58 | 0.06 | 0.205 | 0.615 | - | 0.439 | - | 0.0954 | 0.118 | 1.78 | - |
| **Jericho 5** | 0,.99 | 0.031 | 0.89 | BDL | 0.01 | BDL | BDL | BDL | -2.53 | 0.276 | 0.527 | 0.441 | -2.96 | 1.79 | 2.36 | -7.44 | 0.642 |
